# Predicting the alternative conformation of a known protein structure based on the distogram of AlphaFold2

**DOI:** 10.1002/qub2.70062

**Published:** 2026-11-06

**Authors:** Jiaxuan Li, Zefeng Zhu, Chen Song

**Affiliations:** ^1^ Center for Quantitative Biology Academy for Advanced Interdisciplinary Studies Peking University Beijing China; ^2^ College of Life Sciences Yantai University Yantai China; ^3^ Peking‐Tsinghua Center for Life Sciences Academy for Advanced Interdisciplinary Studies Peking University Beijing China

**Keywords:** alternative conformation, deep learning, distogram, multi‐state protein, protein structure prediction

## Abstract

AlphaFold2 (AF2) has become one of the most established and leading tools for predicting protein structures, although it often favors a single conformation. While it has been shown that sampling different conformations is possible by manipulating the multiple sequence alignment (MSA), the potential for multi‐state information within a standard AF2 prediction remains underexplored. In this work, we demonstrate that even with a deep MSA, AF2 predictions encode structural heterogeneity. Specifically, we observe that the predicted residue–residue distance distributions often exhibit multiple probability peaks before recycling, suggesting the presence of alternative states. We derive state‐specific distance restraints by fitting and separating these multi‐peak distributions. These restraints are then incorporated into Rosetta as energy terms to model large‐scale conformational transitions. Across multiple protein systems undergoing diverse types of conformational changes, our method effectively samples alternative conformations starting from an input conformation, producing models comparable to those from existing approaches. Our findings highlight a previously underutilized feature of AF2 predictions and offer a novel strategy to take advantage of these insights to study conformational flexibility and dynamics in proteins. This work broadens the applicability of AF2 in structural biology and the modeling of multi‐state protein systems.

## 1 INTRODUCTION

Proteins present versatile functional conformation states to execute the biological activities essential for living systems [1, 2]. Modeling protein structures in silico has been a pivotal biological challenge studied for decades. Thanks to advancements in deep learning, accurately predicting protein structures from sequences has become commonplace. Presently, coevolution‐based techniques represent the cutting edge, with AlphaFold2 (AF2) [3] standing among the foremost prediction models. The state‐of‐the‐art methods, such as AF2, RoseTTAFold, and trRosettaX, have significantly improved the prediction accuracy of protein structures, often reaching an accuracy comparable to experimental results [4, 5, 6]. These deep‐learning‐based methods have also facilitated other related studies, such as utilizing predicted structures as AI‐augmented inputs for molecular dynamics simulations [7, 8].

Furthermore, the advent of AF2 has sparked renewed inspiration in predicting multi‐state conformations, which is crucial for comprehending dynamic behaviors and exploring the conformation space from the structural biology perspective [9, 10, 11, 12]. Nonetheless, determining multiple functional states of proteins, such as the apo and holo states of enzymes and the outward/inward‐facing states of transporter proteins, remains challenging both experimentally and computationally. Current prediction methods, including AF2, primarily focus on predicting one particular state [13]. Predicting alternative conformations and assessing the impact of sequence mutations still pose difficulties with standard AF2 usage [14, 15]. Starting from CASP15, prediction targets encompassed ensembles of protein conformations [16], and there is growing interest in developing computational approaches to predict alternative conformations in the field.

There have been many attempts targeting multi‐state conformation predictions through AF2 with certain modifications. It is realized that one can further increase AF2’s sampling diversity via enabling the dropout layers and switching random seeds [17]. However, this approach only contributes subtle changes to the default predictions. Manipulation of multiple sequence alignment (MSA) offers great potential for predicting multiple conformations. The prevalent strategy involves MSA‐subsampling, as reducing MSA depth can introduce greater uncertainty into AF2’s predictions [18, 19]. It has effectively sampled alternative conformational states of transporters by randomly subsampling MSAs down to just 16 sequences. However, this line of work may compromise prediction quality or heavily rely on known structural templates [18]. Notably, Wayment‐Steele et al. successfully extracted the evolutionary signals from different states of metamorphic proteins by clustering the MSAs [20]. Replacing the homology search algorithm and increasing the quality of homologous sequences also contribute to the prediction of relative state populations [21]. The sensitivity of AF2 predictions to variations in MSAs can be further investigated through sequence mutation analysis, performed either column‐wise [22] or row‐wise [23], enabling effective sampling of alternative conformations and putative transitions. There have also been attempts to infer Boltzmann weights from AF2 predictions [7, 24]. More interestingly, several recent deep‐learning‐based methods used generative models to learn the distribution of multiple conformations [25, 26], being a promising direction. Apart from these, another line of work has successfully incorporated explicit ligand conditions to predict the holo‐states of proteins [27, 28, 29].

In a recent study, we demonstrated that deep‐learning‐based models can de novo capture multi‐state information of a protein structure, and proposed a protocol to model the alternative conformation from a known structure based on contact map predictions [30]. In this paper, we further explore multi‐conformation prediction methods by using AF2 and predominantly focus on predicting the alternative conformations of two‐state proteins. We investigate the possibility of accessing these alternative conformations directly from AF2 predictions, without resorting to MSA depth reduction. Starting from an initial structure, we incorporated restraints derived from the probabilistic distribution of distance maps (distogram) of AF2 and successfully achieved satisfactory predictions of the alternative state for multiple two‐state proteins.

## 2 RESULTS

### 2.1 AF2 predictions with full MSA contain alternative conformational information

We first examined 20 proteins known to exist in two distinct structural states. These proteins range in size from 83 to 524 amino acids and encompass distinct conformational changes upon state transitions, including rearrangements of local secondary structures in single‐domain proteins, domain opening, domain rotations, and combinations of complex alterations in multi‐domain proteins. As shown in Table 1, we used template‐based modeling scores (TM‐scores) and root‐mean‐square deviation (RMSD) to evaluate the conformation similarity (${\text{TM}}_{2\text{states}}$) and difference (${\text{RMSD}}_{2\text{states}}$), respectively. More details about the chosen proteins are elaborated in the Materials and Methods section. The average ${\text{TM}}_{2\text{states}}$ of 20 proteins is 0.69, indicating significant differences in conformational states. For consistency, we categorized these proteins into conformation 1 (C1) and conformation 2 (C2) in the representation of their two states, whose experimental structure information are listed in Supporting Information S1: Table S1. We designated the apo state as C1 and the holo state as C2 and followed the definitions established in prior research for some membrane proteins, whose substrate‐bound or inward‐facing state is usually defined as C2 [31, 32, 33, 34].

**TABLE 1:** AlphaFold2 prediction results for 20 selected two‐state protein systems exhibiting significant conformational changes.

Protein	Sequence length	${\text{TM}}_{2\text{states}}$	${\text{RMSD}}_{2\text{states}}$ (Å)	# MSA aligned sequences	${\text{RMSD}}_{\text{AF}2}$ (Å)	
					C1	C2
AK	214	0.69	6.95	17,289	5.69	1.62
LBP	345	0.64	7.14	11,060	6.82	0.64
ALBP	288	0.76	4.45	9168	3.77	1.46
RBP	271	0.76	4.06	9754	3.74	0.54
GroEL	524	0.64	12.90	20,457	11.00	4.38
GlnBP	220	0.67	5.34	9143	5.15	0.76
STP10	480	0.82	4.40	14,446	3.29	1.98
DAHPS	338	0.81	10.10	18,906	9.69	1.32
S100B	83	0.55	5.93	8517	5.55	1.55
CNBD	142	0.74	6.09	10,677	4.92	3.17
NTRC	124	0.64	4.55	17,850	4.32	3.37
AP4A	165	0.80	4.61	13,948	3.64	1.20
48G7g	217	0.57	7.21	12,360	6.30	2.13
S100A1	93	0.50	6.39	10,029	6.06	2.44
T4L	161	0.76	3.33	4078	0.92	2.50
NTnC	90	0.55	6.43	17,122	3.90	4.14
HSPD1	524	0.64	13.20	18,296	3.01	12.70
ZnT8	288	0.70	4.95	11,863	2.96	3.19
Mhp1	463	0.88	3.30	6057	2.30	3.08
ASCT2	427	0.65	10.60	12,605	3.57	7.74

*Note*: Protein length, initial conformational similarity (${TM}_{2\text{states}}$), and difference (${\text{RMSD}}_{2\text{states}}$) between the two states are presented in columns two to four. The fifth column displays the number of sequences aligned using full multiple sequence alignment (MSA). The final two columns show the conformational differences between AlphaFold2 predictions and experimentally solved structures, represented as RMSD values relative to conformation 1 (C1), or conformation 2 (C2).

Abbreviations: AF2, AlphaFold2; RMSD, root mean‐square deviation; TM, template‐based modeling score.

We utilized ColabFold v1.5.2 [3, 17] to predict the structures and obtain the corresponding distograms (DMs) for the selected 20 proteins. Our approach involved a deep MSA without recycling. We hypothesized that accurate protein structure prediction based on enriched coevolutionary information would encompass multiple functional states within the conformational space. By skipping recycling, we preserved the original pair representations from AF2’s Evoformer module and prevented updates that might bias the prediction toward a single, most probable conformation. Analysis of AF2’s default structure predictions revealed a high precision in most models. However, these predictions often favored a single conformational state, aligning with recent findings [9, 13]. This bias is evident in the last two columns of Table 1. For proteins exhibiting significant conformational changes between their two states, such as cyclic nucleotide‐gated potassium channel (CNBD), transcriptional enhancer binding protein (NTRC), and regulatory domain of troponin C (NTnC), AF2’s predictions showed ambiguity. In these cases, the predicted structures deviated substantially from both known states. Given these observations, we sought to extract additional information from AF2’s output. We aimed to separate potential multi‐state information within its distograms, enabling us to predict alternative conformations and potentially enhance the overall prediction accuracy of AF2.

We collected five default AF2 models for each protein, ranked from 001 to 005 based on their predicted local distance difference test (pLDDT) scores, following the protocols outlined in the Materials and Methods (Source of distogram). Our evaluation focused on the precision (Figure 1A) and number abundance (Figure 1B) of alternative conformational signals within the distogram across all 20×5 predicted models. For each of the 20 selected proteins, we analyzed five AF2 models in both prediction directions (from C1 to C2, and from C2 to C1). Our analysis revealed that the rank005 models overall represented the highest precision and abundance of signals associated with alternative conformations. Remarkably, even when the final predicted structures closely resemble a specific conformation, the rank005 distograms still contained significant information about alternative conformations. As illustrated in Figure 1A, rank005 models exhibited the highest precision among rank001–005 models for 60% of the selected proteins. For example, adenylate kinase (AK), d‐allose binding protein (ALBP), leucine binding protein (LBP), and ribose binding protein (RBP) showed high‐precision signals of alternative conformations in rank005 models, whereas other models’ precision fluctuated significantly. Eight proteins—NTnC, human mitochondrial chaperonin (HSPD1), S100 calcium binding protein (S100B), DAHP synthase (DAHPS), zinc transporter 8 (ZnT8), neutral amino acid transporter (ASCT2), S100 metal binding protein (S100A1), and Ap4A hydrolase (AP4A)—demonstrated higher precision in models other than rank005. However, when considering the ratio of signal numbers between other models and rank005 (Figure 1B), except for DAHPS, seven of these proteins showed fewer or more sparsely distributed signals compared to rank005. This analysis suggests that a slight trade‐off in precision may enhance the likelihood of capturing multiple conformations. Rank005 models performed well in capturing alternative conformation signals when considering both precision and quantity, and this came at the cost of marginally lower model confidence, reflected in the pLDDT values (Figure 1C). Based on these results, we chose to use rank005 models for all tested proteins in subsequent analyses. These results highlight the importance of examining lower‐ranked models in AF2 predictions, as they may contain valuable information about alternative protein conformations that might be missed in higher‐ranked models.

**FIGURE 1:**
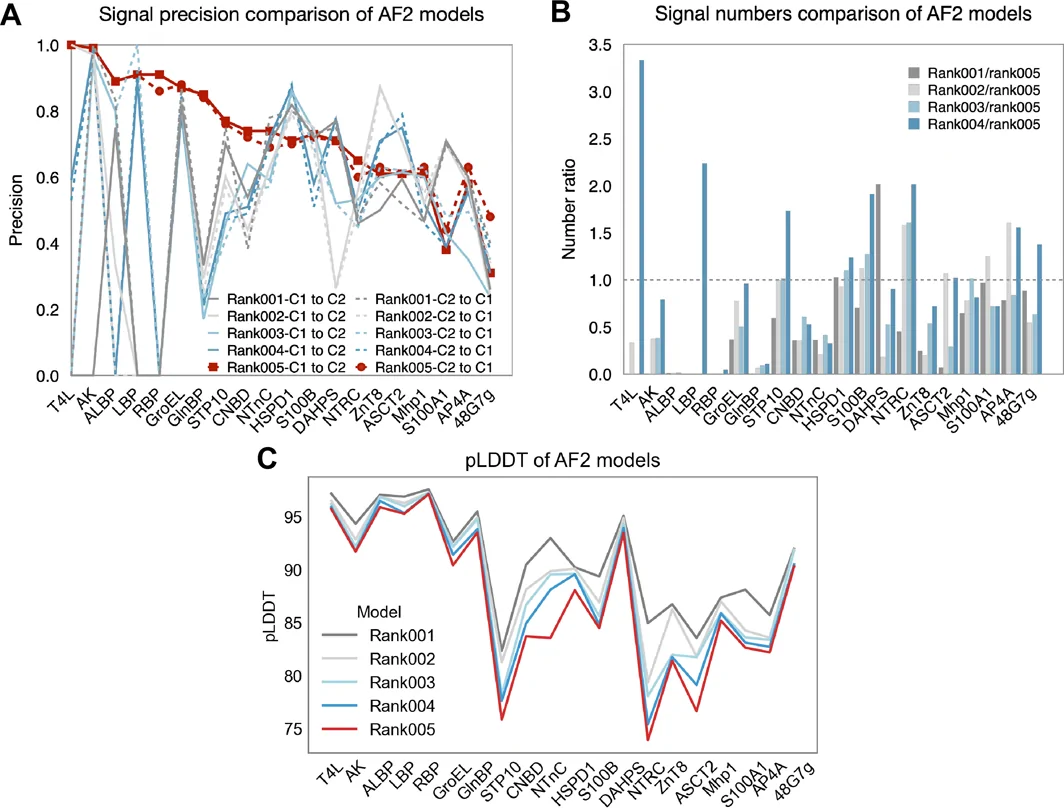
Evaluation of alternative conformation signals within the distogram and model quality of AlphaFold2 (AF2) models without recycling. (A) Precision of alternative conformation signals for the default five models predicted by AF2. Overall, models rank005 demonstrate the best performance in multi‐state prediction. Solid lines represent comparisons to conformation 1 (C1), whereas dotted lines indicate comparisons to conformation 2 (C2). (B) Ratio of alternative conformational signals predicted by AF2 between rank001–004 and rank005 models. Most ratios fall below 1.0, suggesting that rank005 usually captures the most abundant signals of alternative conformations. (C) The predicted local distance difference test (pLDDT) values of the AF2 models from rank001 to rank005.

### 2.2 Extracting structural information of the alternative conformation based on a known structure and AF2 distogram

To utilize the alternative conformational information within AF2’s predictions, we developed a protocol for extracting multi‐peak distribution signals. This method relies on distogram predicted by AF2 and a known initial structure, as illustrated in Figure 2. The initial structures can be experimentally determined structures or any predicted structures, such as the predicted structures directly obtained by AF2. We used AK as an example to demonstrate this process. AF2’s default predicted structure for AK (shown in yellow) closely resembles the C2 (holo‐state) conformation. However, we delved into AK’s predicted distogram and uncovered numerous multi‐peak distance‐probability signals. These signals indicate distance variations between the same residue pairs, suggesting the presence of alternative conformations. By comparing the multi‐peak signals from the AF2 prediction with the initial structure, we can extract distinct distance distributions. These distributions then serve as guidance for predicting the second state.

**FIGURE 2:**
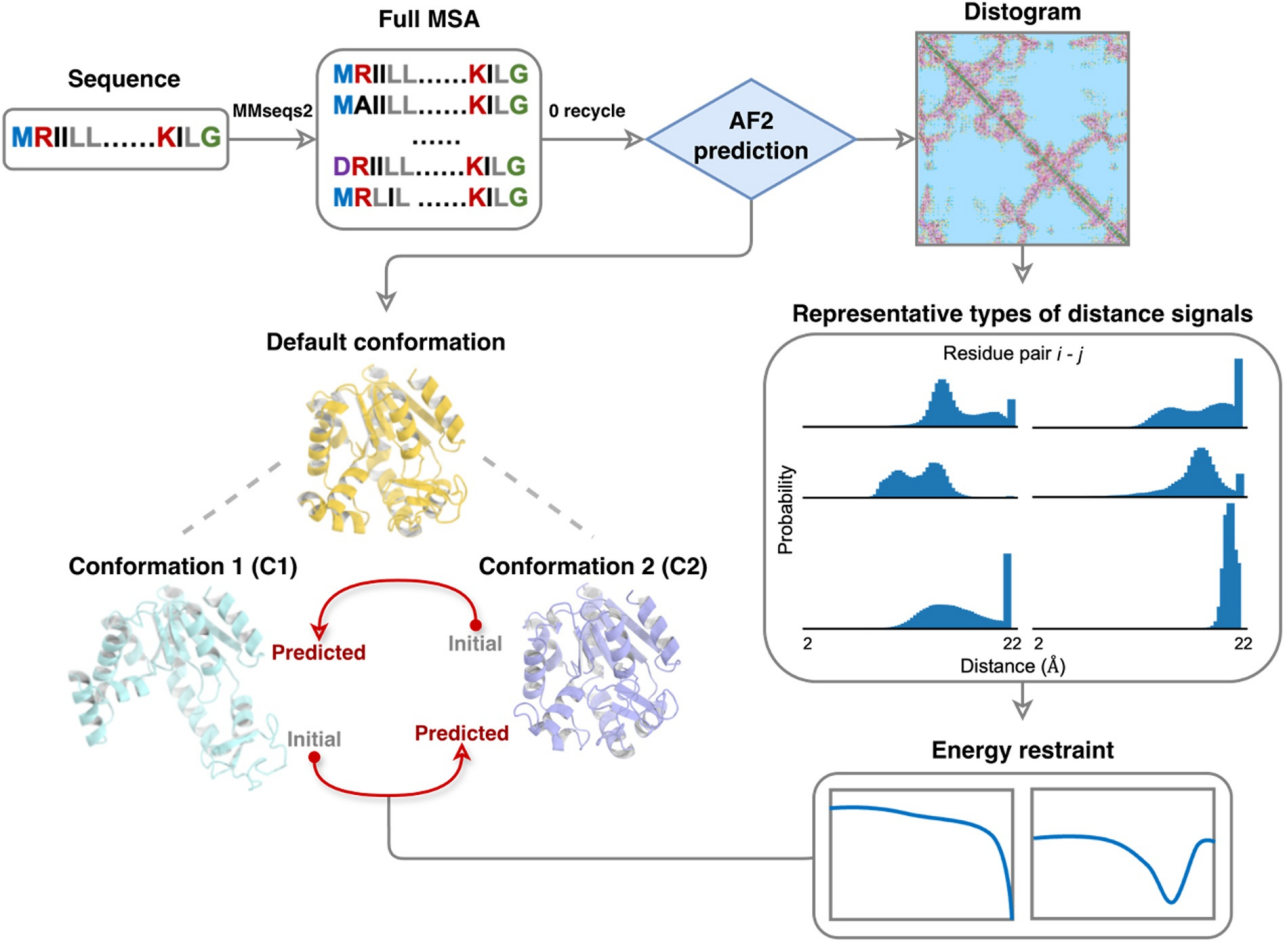
Workflow for predicting alternative conformations from a known structure and AlphaFold2 (AF2) distogram. AF2 was used to predict the default conformation and its distogram, employing deep multiple sequence alignment (MSA) and zero recycle parameters. Starting from one state, we extracted and utilized multi‐peak distance probability signals to predict the alternative state, enabling transitions from conformation 1 (C1) to conformation 2 (C2) and vice versa.

Our method for extracting alternative conformational information involves a detailed analysis of the distance probability distributions in AF2’s predictions. For residue pairs containing multi‐peak signals, we removed the distributions corresponding to the initial state and rescaled the remaining distribution to a total probability of 1. Signals satisfying both conformational states were retained. These processed signals were combined to create a distogram for the alternative conformation (alt‐distogram), which was utilized in designing the restraint energy functions during the modeling process and guided our modeling of the second conformational state.

We categorized the distance‐probability distributions into two main groups: (1) Multi‐peak distributions, suggesting potential alternative distances between residue pairs and indicating conformational changes; (2) Single‐peak distributions, representing stable structural elements across conformational states. We categorized six types of multi‐peak distributions and the typical single‐peak signals. Figure 3 provides a comprehensive visualization of all these signal types. Detailed selection criteria are described in the Materials and Methods section. In brief, our signal extraction process focused on three aspects: (1) Fitting multi‐peak distributions using a bivariate Gaussian distribution (2) Employing the last bin’s data in distogram (comprising 64 equal‐width bins spanning from 2 to 22 Å) as an indicator of long‐distance signals. (3) Incorporating single‐peak signals that differ significantly from the initial structure.

**FIGURE 3:**
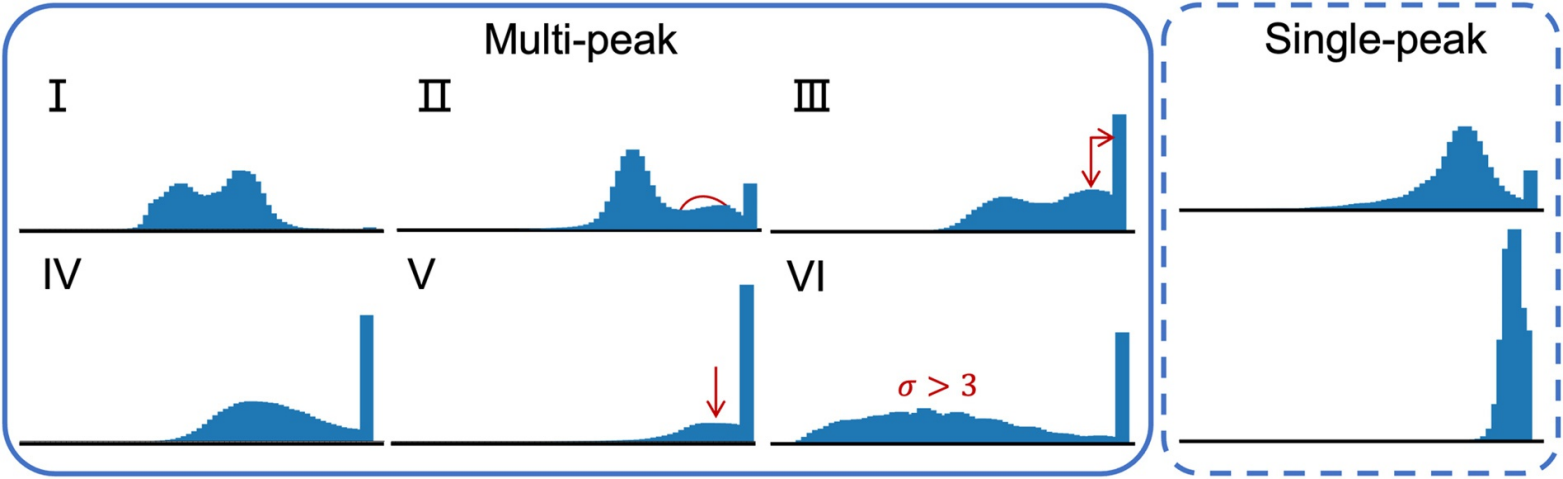
Categorization of distance‐probability distributions. Multi‐peak signals of six specific types and typical single‐peak signals are illustrated. The corresponding selection criteria and features corresponding to the arrows are described in the Materials and Methods section.

As is evident from Table 2, AF2’s predictions for most of the studied proteins revealed an abundance of hidden signals of the alternative conformation. We analyzed the number of true positive and false positive multi‐peak signals (# Multi‐peak), as well as the single‐peak signals distinct from any known state (# Single‐peak). For 12 out of 20 proteins, AF2 predictions with deep MSA yielded hundreds of multi‐peak signals, potentially valuable for modeling alternative conformations. An interesting outlier was T4 lysozyme (T4L), which lacked multi‐peak signals but contained four significant single‐peak signals distinct from the known structure. Moreover, our signal selection protocol demonstrated remarkable precision in multiple instances, above 0.85 for several multi‐domain proteins. The average alternative conformation signal precision was 0.73 across the 20 proteins, effectively pinpointing the key residue pairs associated with the conformational differences between the two states.

**TABLE 2:** Analysis of alternative conformation signals from AF2 distogram for 20 selected two‐state proteins.

Protein	# Multi‐peak		# Single‐peak				Precision	
	TP	FP	${\text{TP}}_{\text{C1}}$	${\text{FP}}_{\text{C1}}$	${\text{TP}}_{\text{C2}}$	${\text{FP}}_{\text{C2}}$	C1	C2
AK	386	3	37	0	48	0	**0.99**	**0.99**
LBP	433	46	75	3	53	3	**0.91**	**0.91**
ALBP	272	34	12	0	5	0	**0.89**	**0.89**
RBP	12	2	8	0	0	0	**0.91**	**0.86**
GroEL	333	41	61	17	74	17	**0.87**	**0.88**
GlnBP	202	30	16	9	8	9	**0.85**	**0.84**
STP10	646	187	89	34	70	34	**0.77**	**0.76**
DAHPS	141	56	6	4	3	4	**0.71**	**0.71**
S100B	20	7	4	2	3	2	**0.73**	**0.72**
CNBD	50	20	35	10	27	10	**0.74**	**0.72**
NTRC	27	18	21	8	12	8	**0.65**	**0.60**
AP4A	73	42	20	25	40	25	**0.58**	**0.63**
48G7g	11	28	11	20	34	20	**0.31**	**0.48**
S100A1	8	12	3	6	6	6	**0.38**	**0.44**
T4L	0	0	3	0	1	0	**1.00**	**1.00**
NTnC	28	8	29	12	16	12	**0.74**	**0.69**
HSPD1	287	117	73	33	65	33	**0.71**	**0.70**
ZnT8	269	192	41	10	77	10	**0.61**	**0.63**
Mhp1	329	170	56	79	99	79	**0.61**	**0.63**
ASCT2	834	551	123	50	121	50	**0.61**	**0.61**

*Note*: The table quantifies signals that differ from the known state, categorized as multi‐peak (# Multi‐peak), and single‐peak (# Single‐peak) distributions. Precision scores, as defined in Equation (3), are calculated based on known conformation 1 (C1 precision) and known conformation 2 (C2 precision). The bold font was intended to emphasize the core results.

Abbreviations: FP, false positive; TP, true positive.

### 2.3 Modeling the alternative conformation with Rosetta

We further investigated whether the distance information identified above could aid in predicting the alternative state. With either C1 or C2 as the initial state, we constructed models for the alternative conformations of all 20 proteins in two directions (C1 to C2, or C2 to C1). This conformational exploration can be tackled through a physics‐based optimization approach with the widely used Rosetta software suite (2019.42+release.3666509).

#### 2.3.1 Converting distogram into energy restraints for Rosetta modeling

The distance‐probability distributions within distograms were converted into distance‐based restraint energy functions and incorporated into Rosetta’s energy function during energy minimization and full‐atom refinement stages. We constructed energy–distance functions (Figure 4) based on the alternative distogram (alt‐distogram) to guide the initial conformation toward the alternative state. Our sampling framework, which includes structure initialization, energy minimization, and full‐atom refinement stages, resembles the protocol described in our recent work [30].

**FIGURE 4:**
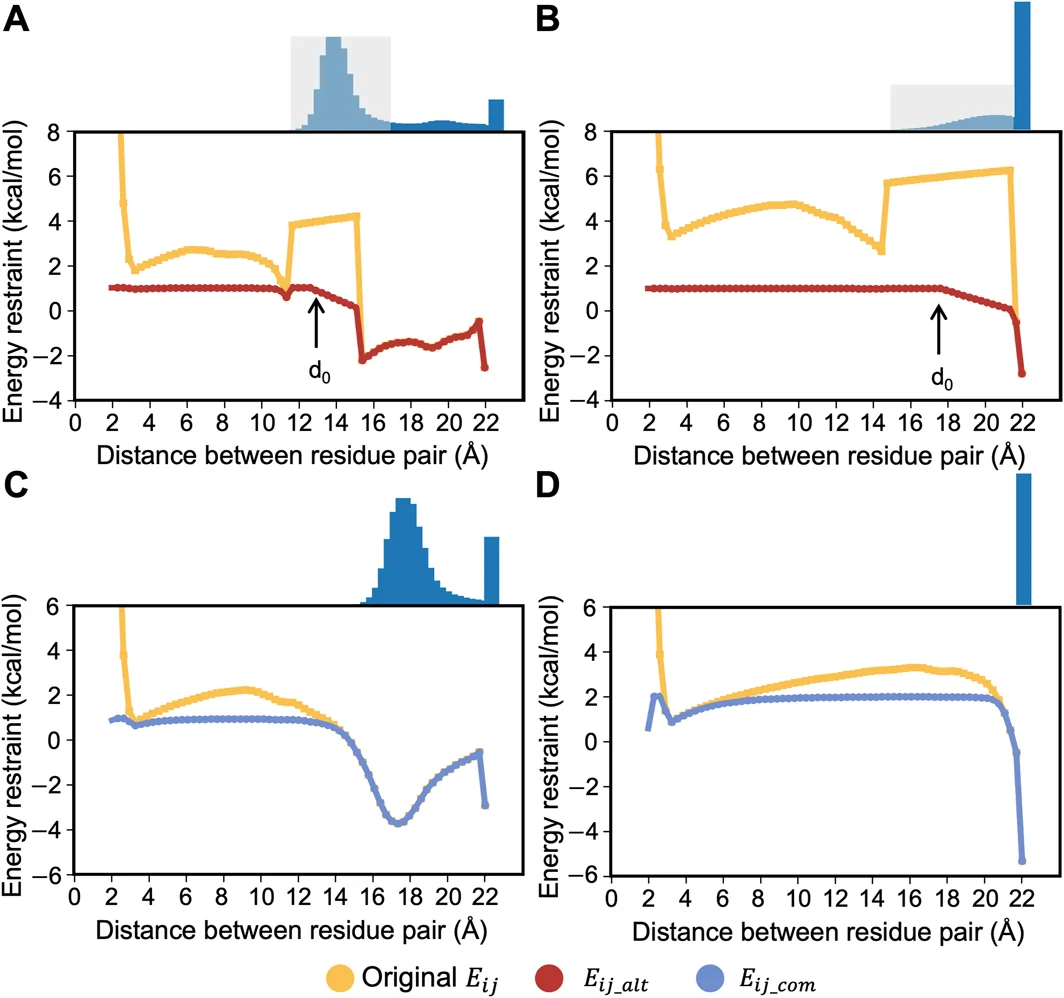
Restraint energy function for residue pairs with alternative (A, B) or common distributions between two states (C, D). Distance distributions from 2 to 22 Å correspond to blue signals at the top of each panel, with gray areas covering distance intervals specific to the initial state. The high energy barrier in the original distribution (yellow) is removed and smoothed in the designed energy function (red for $E_{ij\_\text{alt}}$ and blue for $E_{ij\_\text{com}}$). (A) Alternative state: predicted distance within the first 63 bins; initial distance in the known state ($d_{0}$) = 12.7 Å. (B) Alternative state: predicted distance over 22 Å (last bin); $d_{0}$ = 17.8 Å. (C) Common distribution: concentrated in the 16–18 Å range. (D) Common distribution: concentrated in the last bin (>22 Å).

We distinguished between distance signals of alternative conformations ($E_{ij\_\text{alt}}$, Figure 4A,B) and common signals shared by the two conformational states ($E_{ij\_\text{com}}$, Figure 4C,D) in the restraints. As shown in Figure 4, the energy barrier (yellow lines) associated with the known structure is flattened, facilitating changes in inter‐residue distances during conformational transitions. For instance, the initial distance ($d_{0}$) between the residue pair is 17.8 Å for Figure 4B, whereas its predicted distance in the second state exceeds 22 Å, corresponding to the high probability distribution signal in the last bin.

A scaling parameter (mag) was used to balance the restraint strength between sparse alternative signals and common signals shared between two states. Residue pairs with precise distance predictions (lower entropy H) were assigned higher weights, whereas signals with ambiguous distance predictions (higher entropy H) were given less emphasis in the restraints. Signal density ($p_{nl}$), defined as the ratio of alternative signals to sequence length, and the proportion of signals associated with the last bin among all alternative signals ($p_{\text{last}‐\text{bin}}$) were also considered in the parameter design. This approach aimed to effectively magnify restraints for alternative distributions while maintaining the overall structural rationality. For more detailed information, please refer to the “Structural modeling with Rosetta” subsection in the Materials and Methods section.

#### 2.3.2 Assessment of predicted structures

After incorporating energy functions into the Rosetta modeling process, we sampled 150 models for each state of the 20 selected protein systems, using either C1 or C2 as the initial structure.

The sampling results, evaluated using Rosetta energy scores and TM‐scores relative to each experimental structure, are displayed in Supporting Information S1: Figures S1 and S2. We established criteria for selecting the best‐scored models from a prediction standpoint (detailed in the Materials and Methods section). Priority was given to models exhibiting relatively lower energy scores and significant variations in TM‐score compared to the initial structures. The top 10 models for each prediction, selected according to these criteria, are highlighted in the zoomed‐in views, with colors ranging from blue to red. The blue point represents the best‐scored model, and its TM‐scores relative to C1 and C2 are labeled in each inset. It should be noted that these criteria were developed using the AK, ALBP, DAHPS, and ZnT8 proteins as model systems, the remaining proteins being used for split testing to avoid parameter overfitting.

The quantitative prediction performance of our method is presented in Table 3, showcasing both the best‐scored and best‐sampled (best prediction among all samples) results for the two prediction directions. We considered a prediction successful if the TM‐score relative to the target conformation exceeded the TM‐score relative to the initial state. Successful predictions meeting this criterion are emphasized in bold font. When predicting from C1 to C2, the success rate was 85% (17/20) for the best‐scored models and 90% (18/20) for the best‐sampled models. In the opposite direction, from C2 to C1, the success rate was slightly lower at 70% (14/20) for both cases. C2 is the AF2‐biased state for the 14 systems studied, which explains why the prediction from C1 to C2 behaved better. The alternative state, C1, is more difficult to predict. Proteins with limited alternative signals (T4L) and significant conformational differences (${\text{TM}}_{2\text{states}}\leq 0.55$, S100B, S100A1, NTnC) posed greater challenges for predictions. The unsatisfactory TM‐score performance of ASCT2 could be attributed to false‐positive signals in the last bins.

**TABLE 3:** Evaluation of predicted alternative states for 20 selected proteins.

Protein	${\text{TM}}_{2\text{states}}$	TM‐score (C1 to C2)				TM‐score (C2 to C1)			
		${\begin{array}{c} \text{Best}\;\\ \text{scored} \end{array}}^{C1}$	${\begin{array}{c} \text{Best}\;\\ \text{scored} \end{array}}^{C2}$	${\begin{array}{c} \text{Best}\;\\ \text{sampled} \end{array}}^{C1}$	${\begin{array}{c} \text{Best}\;\\ \text{sampled} \end{array}}^{C2}$	${\begin{array}{c} \text{Best}\;\\ \text{scored} \end{array}}^{C1}$	${\begin{array}{c} \text{Best}\;\\ \text{scored} \end{array}}^{C2}$	${\begin{array}{c} \text{Best}\;\\ \text{sampled} \end{array}}^{C1}$	${\begin{array}{c} \text{Best}\;\\ \text{sampled} \end{array}}^{C2}$
AK	**0.69**	0.71	**0.93**	0.71	**0.94**	**0.88**	0.73	**0.91**	0.73
LBP	**0.64**	0.66	**0.99**	0.66	**0.99**	**0.93**	0.65	**0.93**	0.65
ALBP	**0.76**	0.78	**0.96**	0.78	**0.96**	**0.86**	0.70	**0.93**	0.75
RBP	**0.76**	0.77	**0.99**	0.77	**0.99**	**0.89**	0.85	**0.90**	0.83
GroEL	**0.64**	0.64	**0.78**	0.65	**0.88**	**0.78**	0.66	**0.80**	0.69
GlnBP	**0.67**	0.64	**0.98**	0.65	**0.98**	**0.72**	0.60	**0.84**	0.71
STP10	**0.82**	0.85	**0.94**	0.84	**0.95**	**0.92**	0.87	**0.93**	0.88
DAHPS	**0.81**	0.79	**0.90**	0.80	**0.95**	**0.81**	0.81	**0.83**	0.80
S100B	**0.55**	0.58	**0.81**	0.60	**0.81**	0.57	0.75	0.62	0.76
CNBD	**0.74**	0.70	**0.86**	0.70	**0.86**	**0.74**	0.70	**0.80**	0.72
NTRC	**0.64**	0.65	**0.73**	0.68	**0.75**	0.66	0.74	0.69	0.75
AP4A	**0.80**	0.82	**0.86**	0.82	**0.86**	**0.81**	0.77	**0.83**	0.83
48G7g	**0.57**	0.56	**0.85**	0.58	**0.87**	**0.70**	0.57	**0.80**	0.59
S100A1	**0.50**	0.52	**0.72**	0.54	**0.74**	0.51	0.71	0.54	0.73
T4L	**0.76**	0.92	0.85	0.92	0.85	**0.97**	0.80	**0.97**	0.80
NTnC	**0.55**	0.46	**0.62**	0.54	**0.68**	0.60	0.63	0.64	0.65
HSPD1	**0.64**	0.59	**0.70**	0.63	**0.73**	0.65	0.66	0.70	0.79
ZnT8	**0.70**	0.62	0.66	0.75	**0.83**	**0.80**	0.74	**0.81**	0.74
Mhp1	**0.88**	0.90	**0.91**	0.91	**0.91**	**0.93**	0.85	**0.95**	0.87
ASCT2	**0.65**	0.61	0.53	0.88	0.62	0.59	0.55	0.66	0.82

*Note*: The initial conformational similarity between two states is represented by ${\text{TM}}_{2\text{states}}$. Best‐scored and best‐sampled results are listed for both prediction directions. Superscripts denote the reference conformation for TM‐score calculations. Successful predictions, defined as those closer to the target state than the initial structure, are highlighted in bold.

The prediction capability of our protocol for alternative conformations was demonstrated across proteins of various sizes and conformational changes, as illustrated in Figures 5 and 6. Figure 5 provides structural visualizations, showing AF2’s predicted models in yellow, representative experimental C1 structures in gray, and C2 structures in blue. The predicted C1 (green) and C2 (purple) structures are aligned to their target states, respectively, visually demonstrating the prediction precision. For proteins exhibiting relatively simple changes, such as domains “opening” and “closing” through hinge‐bending (LBP, ALBP, RBP), we achieved an average TM‐score of 0.98 for C2 and 0.92 for C1. In these cases, AF2 provided remarkably accurate predictions closely resembling C2, whereas our successful modeling of C1 was highly valuable, predicting the alternative conformation not directly provided by AF2. More interestingly, for proteins involving domain rotation (DAHPS, immunoglobulin 48G7 germline Fab‐48G7g) and complex conformational changes (AK, 60 kDa chaperonin‐GroEL), the effectiveness of the designed energy restraints can be inferred from valid predictions in both directions as well. For example, the initial TM‐score between the two states of 48G7g is 0.57, indicating a major conformational difference through domain rotations (Figure 5). Our predicted C1 and C2 structures achieved TM‐scores of 0.80 and 0.87 relative to the experimental structures, respectively, correctly capturing the rotation tendency between domains while maintaining accurate structure prediction within single domains. However, in certain challenging cases (e.g., ASCT2, S100A1), our method proved less efficient in rearranging helices due to limited precision and sparse signals from the alt‐distogram analysis.

**FIGURE 5:**
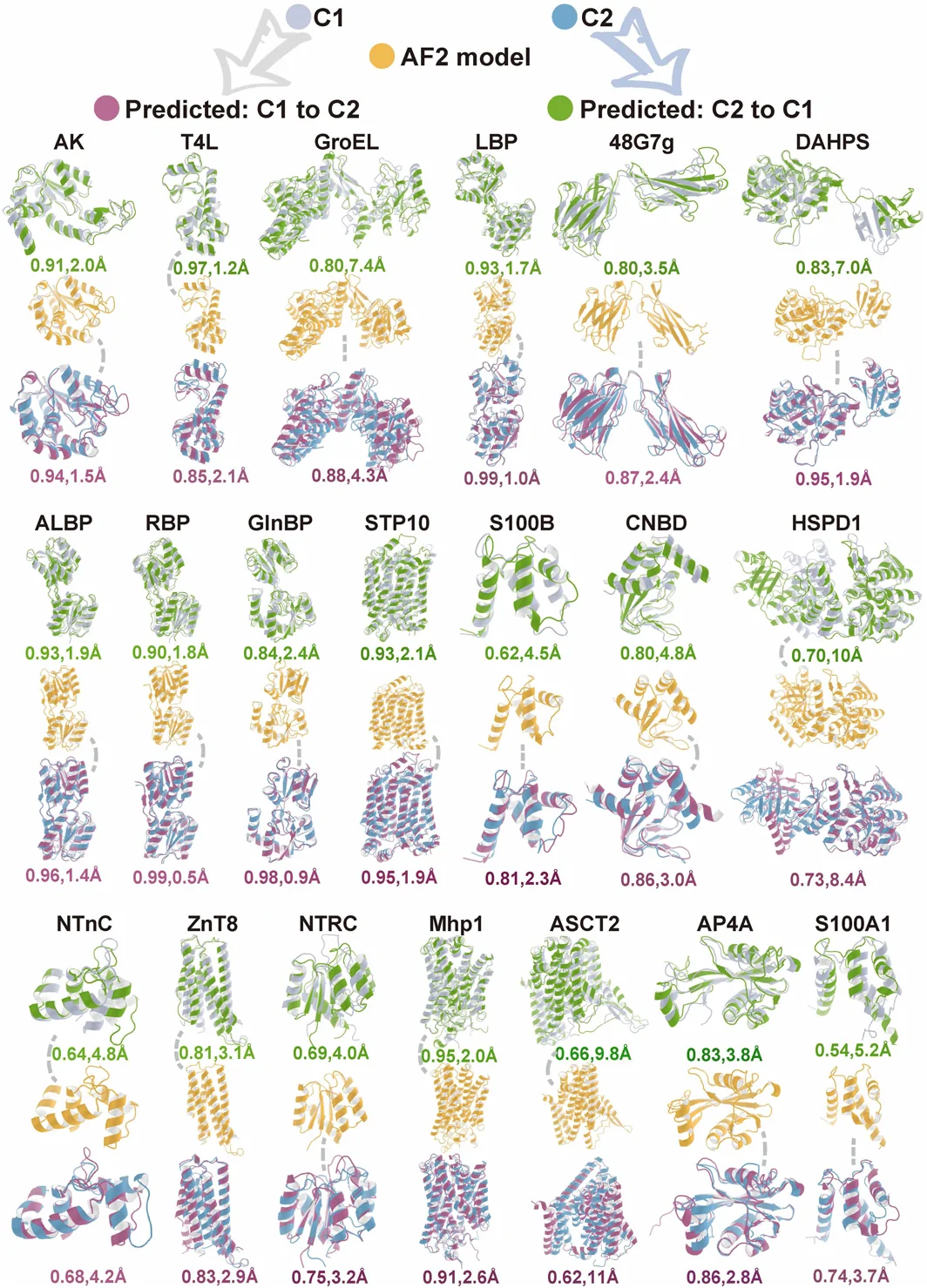
Predictions for the 20 selected proteins with two conformational states. Initial experimentally solved structures (C1: gray; C2: blue) are aligned with best‐sampled structures (predicted C1: green; predicted C2: purple). The default AF2 models (yellow) are shown in the middle rows, connected by dotted lines to conformations with higher similarity. TM‐scores and RMSDs for predictions are shown in color‐coded boxes (green for C1, purple for C2). Nine out of twenty proteins achieved TM‐scores above 0.90.

**FIGURE 6:**
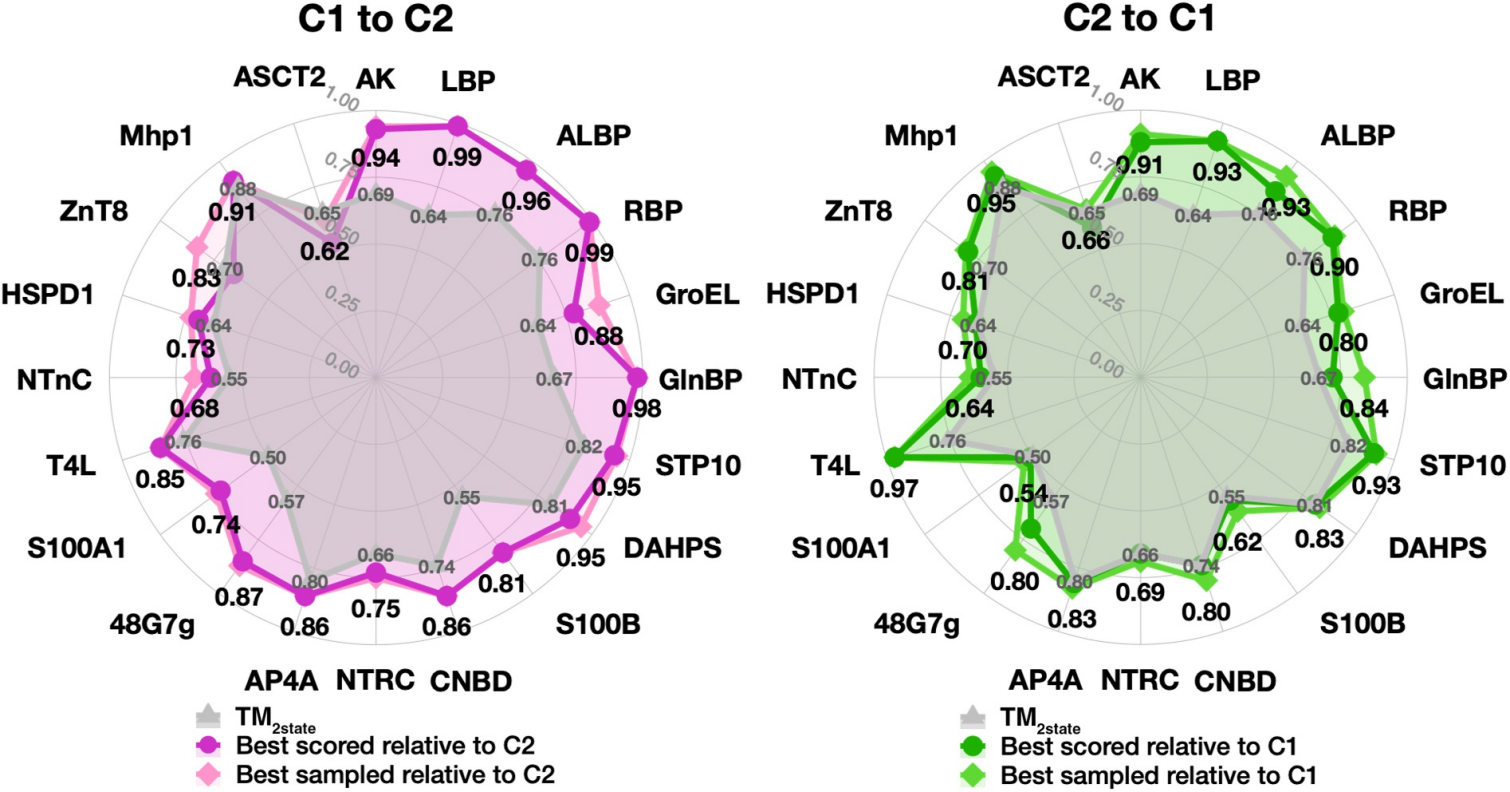
Radar maps of TM‐scores for the 20 proteins in bidirectional predictions. C1 and C2 represent conformations 1 and 2, respectively. The gray region indicates the initial conformational similarity (${\text{TM}}_{2\text{states}}$) between the states. Colored shapes show the TM‐scores between the predicted structures and the target state, with darker‐colored borders representing the best‐scored model and lighter‐colored borders the best‐sampled model.

Figure 6 presents a radar map of the TM‐score results for the 20 proteins. The gray area indicates the initial conformation similarities between the two states, whereas the best‐scored (darker colored lines) and best‐sampled (lighter colored lines) results in both prediction directions cover larger areas, demonstrating that the modeling successfully steered the structure from one state toward the other. For the prediction from C1 to C2, our predicted structures became closer to the target C2 conformation in 19 out of 20 systems (ASCT2 being the exception), achieving high precision (TM‐score > 0.90) in eight protein systems. Even in the more challenging direction (C2 to C1), where most target conformations deviate significantly from AF2’s default output models, we successfully predicted conformations approaching C1, with seven proteins reaching a TM‐score over 0.90. The average TM‐scores for predictions in the C1 to C2 and C2 to C1 directions were 0.86 and 0.80, respectively, across the 20 protein systems. These results demonstrate the effectiveness of our protocol in modeling two‐state proteins with various types of significant conformational changes.

We conducted two control experiments to assess the effectiveness of our method. The first control experiment (CTRL1) used the complete AF2 distogram for modeling, without our signal selection protocol. The second control experiment (CTRL2) omitted the scaling parameter mag in the energy restraint function, treating all signals with equal weights. Supporting Information S1: Figure S3 compares the sampling results of these control experiments with those of our method. Dotted lines depict the TM‐score relative to the initial state in each prediction direction, whereas solid lines represent the TM‐score relative to the target state. Our sampling results exhibited higher precision compared to both control experiments in 18 out of 20 proteins for two directions (as shown by the more peripheral purple and green solid lines). Notably, in the control experiments, the dotted lines were more peripheral, indicating that the predicted conformations were biased toward the initial structures rather than transitioning to the alternative state. This observation supports the effectiveness of our prediction method, which uses selected signals of the alternative conformation and balances the energy function with higher weights for these signals. Detailed results and sampling plots can be found in Supporting Information S1: Tables S2 and S3 and Figures S4–S7.

### 2.4 Distogram‐guided modeling of alternative conformation further refines the AF2 predictions

We wonder whether distogram prediction and analysis can help improve the sampling performance of the different conformation states based on the default AF2 models. To validate it and benchmark against existing approaches, we evaluated the prediction performance using the recently established OC23 and TP16 datasets of AFsample2 [35]. Dataset OC23 comprises 23 open‐closed state proteins, whereas TP16 contains 16 transporter proteins. We obtained the five structure models of AF2 in the setting of deep MSA input with zero recycle, denoted AFzero, and later used each structure model and the corresponding distogram predictions as starting points for Rosetta‐based modeling. Each Rosetta‐based modeling procedure generated 150 models, yielding a total of 750 models per protein system, denoted as RosettaModel. Therefore, the combination of AFzero and RosettaModel is the result of our method and will be used for further analysis.

As shown in Figure 7, AFzero lagged behind the performance of current approaches using only five sampled models per system, compared to 1000. However, Rosetta‐based modeling further refined the predictions with the information obtained from distogram, demonstrating the power of deep MSAs and the inherent structural heterogeneity captured by AF2. When focusing on the performance of the best models, the five AFzero models together with 750 Rosetta models achieve comparable accuracy with existing approaches in predicting alternative conformations, particularly for targets undergoing substantial structural rearrangements. Quantitative analysis also revealed a complementary effect between AFzero and Rosetta samples in both datasets. As quantified by AUC under varying TM‐score thresholds (Figure 7C), our method combining AFzero with RosettaModel achieved the same level of performance as AFsample [36]. Specifically, our distogram‐based modeling approach refines the predictions beyond AFzero for 18 open states and 5 closed states of OC23 targets, and 8 inward‐facing and 4 outward‐facing states of TP16 targets. A representative case is target O76728: although existing methods generated weak open state predictions and AFzero achieved TM‐score = 0.84, our distogram‐based protocol produced a higher‐accuracy model with TM‐score = 0.96 (Figure 8A,B). These results validate the efficacy and robustness of our method in systematically extracting and utilizing the signal of alternative conformations that are embedded within the predicted distogram of AF2, demonstrating the advantages for proteins undergoing large‐scale conformational changes.

**FIGURE 7:**
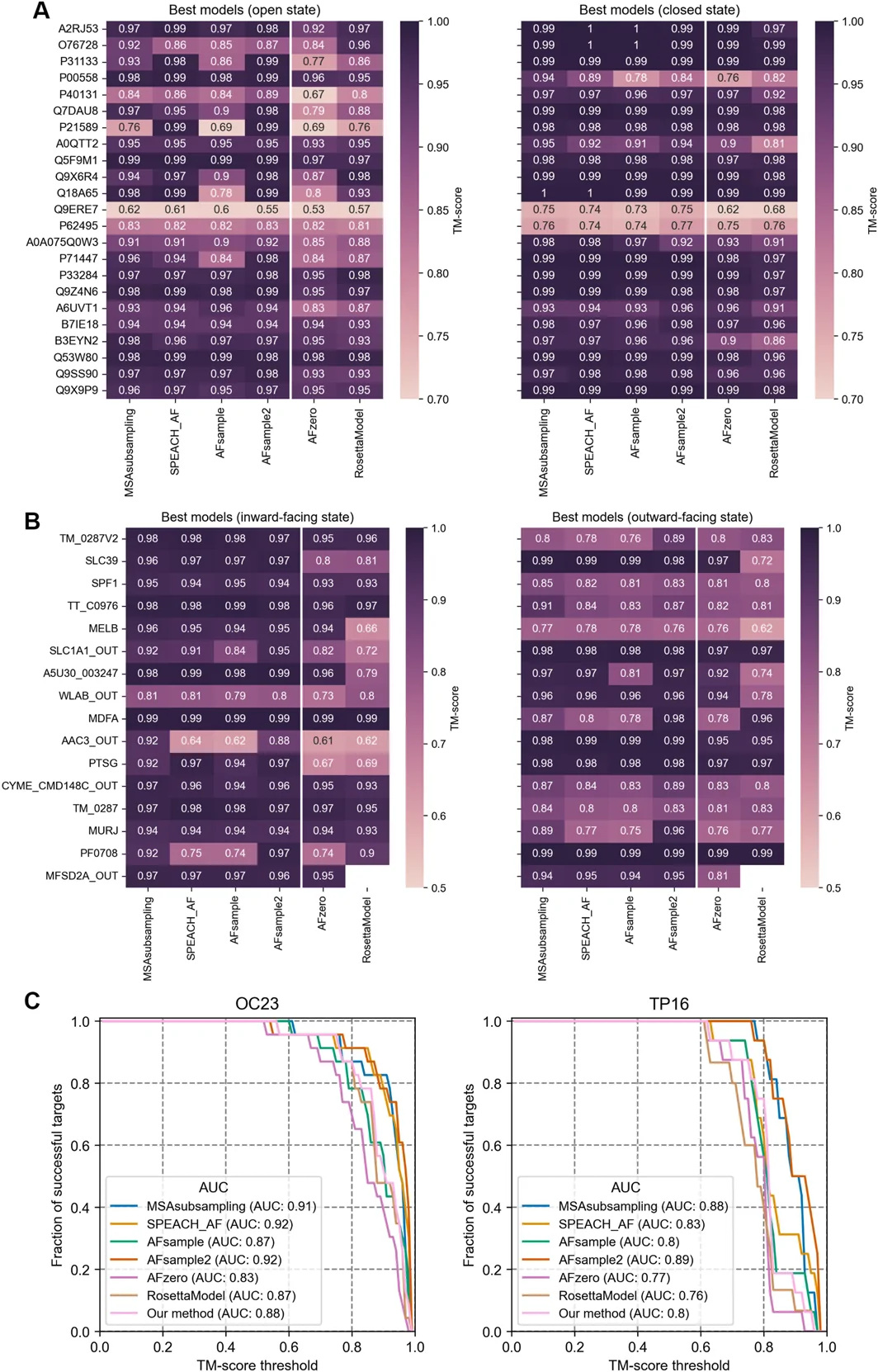
Comparison of our method (combination of AFzero and subsequent RosettaModel) with other recent algorithms in predicting alternative conformations for the two datasets of AFsample2. (A) Comparing TM‐scores of best models generated for open and closed states by all methods with dataset OC23. (B) Comparing TM‐scores of best models generated for transporter proteins by all methods with dataset TP16. For MFSD2A_OUT, no alternative signal was detected and thus no Rosetta model can be generated. (C) Fraction of successful targets compared among methods at different levels of TM‐score thresholds with the OC23 and TP16 datasets. Success: TM‐score > threshold (for both states). AUC, Area Under the Curve. The generated models of previous approaches and the analysis protocol were adapted from AFSample2. TM‐scores were calculated using the default parameters of TM‐align.

**FIGURE 8:**
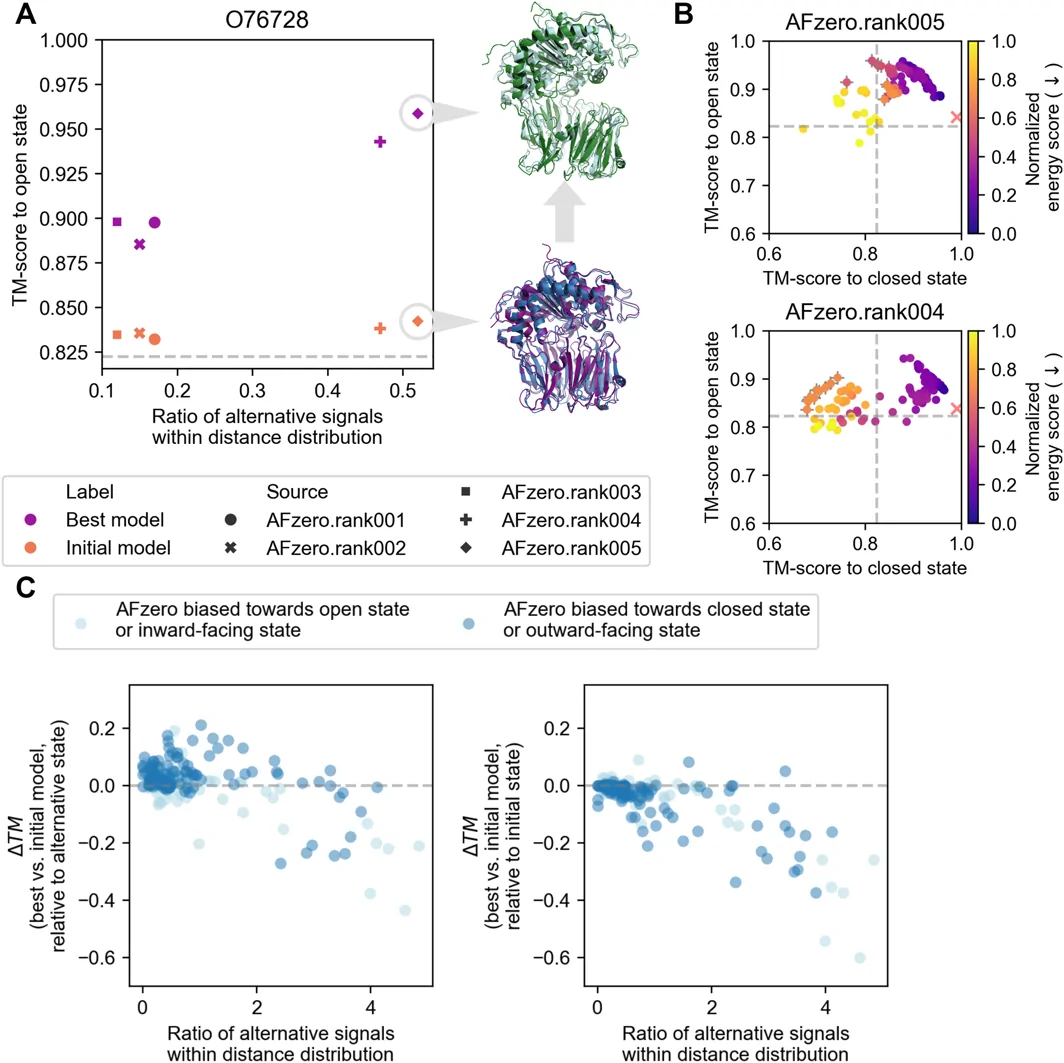
Performance for sampling alternative conformations initiated from AFzero models and guided by the detected alternative signals from the corresponding distogram predictions. (A) The ratio of alternative signals, identified from predicted distogram, is plotted against the TM‐score of the resulting open conformation generated by Rosetta‐based sampling. The source of the initial models and Rosetta samples are indicated by different markers. The initial models and the Rosetta samples are differentiated by different colors. The experimentally solved structures are colored gray and blue. The best‐sampled structures are colored green and purple. (B) Comparison of the Rosetta samples with the open state and the closed state. The Rosetta samples present a diverse distribution away from the initial closed‐state conformation. Based on the TM‐score with the initial state and the normalized energy score denoting the satisfaction of the putative alternative distance restraints, the top 10 models are selected and marked with crosses. The initial structures are marked with red crosses. The dashed line indicates the TM‐score between the open‐state and closed‐state structures. (A, B) O76728 is used as a proof‐of‐concept example. (C) Analysis of the relationship between the ratio of alternative signals in distogram predictions and Rosetta‐based sampling performance, using each of the AFzero predictions for the OC23 and TP16 targets as starting points. Both panels show the greatest changes in TM‐score with distogram‐guided Rosetta modeling. The left panel displays the TM‐score difference relative to the alternative state, whereas the right panel shows the difference relative to the initial biased state. Positive values on the vertical axis indicate improved modeling performance, either toward the alternative conformation or refinement toward the initial state.

However, we also have to note that for the MFSD2A_OUT target within the TP16 dataset, our method did not find any alternative signal from rank001 to rank005’s predicted distogram. And for the MELB, SLC1A1_OUT, and A5U30_003247 targets, the error‐prone signals lead to degenerate Rosetta modeling results, indicating an inherent limitation of our method. A systematic analysis of the 190 models for the OC23 and TP16 targets (38 protein systems except MFSD2A_OUT, with five replicas for each system) revealed that a higher ratio of alternative signals in the distogram does not necessarily lead to better modeling performance (Figure 8C). There are also cases where models neither converge toward either known state nor remain stable, resulting in degeneration. Nevertheless, as long as there are a sufficient number of effective alternative signals, most modeling systems generate samples optimized toward the alternative state, whereas a minority are refined back to the initial state.

## 3 DISCUSSION

In recent years, deep‐learning‐based protein structure prediction has exhibited remarkable accuracy, particularly for simple proteins [3, 4]. A limitation of most current methods is their focus on predicting a specific structure, neglecting the existence of multiple‐state conformations, whereas structural heterogeneity underpins protein functions [37]. However, this limitation does not indicate that deep‐learning methods are incapable of predicting multiple conformations. We believe in their vast potential for this task. The prediction of alternative states is increasingly gaining attention from various research perspectives [13, 18, 21, 22, 25, 30, 35, 38, 39]. In this work, our results revealed that AF2 predictions inherently contain latent information about the alternative structural states for the proteins with multiple functional states.

With this hidden information extracted and combined with Rosetta, we successfully predicted alternative conformations for 19 two‐state proteins starting from either C1 or C2. Subsequently, we achieved performance comparable to AFsample [36] on the OC23 and TP16 datasets, initialized from AFzero’s models and generating 750 Rosetta‐refined decoys (vs. 1000 models used by AFsample). Our method’s effectiveness is evident when the target conformations differ significantly from those predicted by AF2, demonstrating our protocol’s ability to identify and utilize the distance distributions of the alternative conformations for structure modeling. A key distinction of our approach is its orthogonality to MSA‐subsampling strategies [18]. Rather than reducing the depth of the alignment to introduce diversity, we leveraged the full deep MSA to preserve structural fidelity while explicitly extracting latent alternative distance information from the predicted maps. This makes the protocol complementary to existing sampling methods.

However, a crucial factor influencing prediction quality is the initial distogram predicted by AF2. As we rely on AF2 models generated without the recycling procedure, not all test proteins achieved high precision. Ambiguous conformations between two functional states predicted by AF2 can sometimes introduce numerous false signals. For example, the RMSDs of the zinc transporter ZnT8 predicted by AF2 relative to its two states are 2.96 Å and 3.19 Å, which do not correspond to either state. The precision of ZnT8’s alternative signal prediction from two directions both fall below 0.70, further highlighting the challenge of distinguishing between functional conformations in such cases. The presence of false‐positive distributions on structurally important residue pairs, especially those separated by long distances exceeding 22 Å, can adversely affect the precision of the conformation sampling process. Thus, our performance still lags behind the state‐of‐the‐art AFsample2 [35], highlighting room for improvement. Additionally, this study focused exclusively on two‐state protein systems without addressing more complex multi‐state scenarios. One possible approach would be decomposing the multi‐states into two‐state systems and implement multiple distogram generations, using, for instance, different random seeds, and repeating the signal detection procedure as well as the subsequent Rosetta‐based modeling. In fact, a special system, Lipid II flippase MurJ [40], which has been studied in this work, indeed presents multiple conformational states including the inward‐open, inward‐closed, and outward‐facing states. These conformational changes are tested in the OC23 (the B7IE18 target) and TP16 dataset (the MURJ target), respectively. Still, there is room for improvement for modeling the outward‐facing states using our method.

To address limited prediction precision in certain proteins, we anticipate that deep‐learning models other than Rosetta, such as Distance‐AF [41], may enhance the utilization of extracted alternative information from the distograms. Encouragingly, MD‐based enhanced sampling procedures [42, 43, 44, 45] and data curation [46], smarter MSA subsampling strategies [21], and deep‐learning‐based generative models [25] may provide more comprehensive pictures for multi‐state structures and even for the metamorphic proteins (fold‐switchers) that adopt distinct stable folds. Therefore, we are optimistic that the rapid development of the field and probably the combination of the above methods can solve the problem in the near future.

The training procedure and dataset scheduling of AF2 [3] facilitate its capacity to predict multiple conformational states. Most protein systems analyzed were included in the AF2 training dataset; only a few, ZnT8, ASCT2, A0QTT2, Q18A65 (open state), SLC1A1_OUT (inward‐facing state), PTSG (inward‐facing state), MDFA (outward‐facing state), PF0708 (inward‐facing state), AAC3_OUT (inward‐facing state), SPF1, and MELB (inward‐facing state), were deposited in the PDB [47] after 30 April 2018, and lack remote structural homologs with conformations closely resembling the target state in the training set. Nonetheless, our results on these out‐of‐training‐set targets demonstrate that AF2 can generalize its multi‐state prediction capability to novel protein systems. This suggests an avenue for extending this approach to proteome‐wide analyses, aimed at identifying proteins capable of adopting alternative conformations not yet captured by current studies.

To further explore this potential, we extended the analysis to an additional 20 protein systems for which only a single conformational state has been deposited in the PDB, with no alternative models exhibiting pairwise Cα RMSD >2 Å and < 30% sequence identity to any previously investigated system (Supporting Information S1: Table S4 and Figure S8). The same sampling protocol was applied throughout. Our analysis revealed that most modeled protein systems exhibit some degree of conformational flexibility although most sampled conformations closely resemble known PDB structures (Supporting Information S1: Figure S9A). In several cases, our approach uncovered putative alternative conformations. For instance, P41440, a reduced folate transporter with a known inward‐open structure (PDB: 7XQ1), produced samples suggesting potential inward‐closed and outward‐facing states using our protocol (Supporting Information S1: Figure S9B). Although some sampled models appeared degenerate, likely due to suboptimal distance restraints, other cases provided useful inference of functional dynamics. For example, Q5VTY9, a hedgehog acyltransferase, exhibited putative large‐scale fluctuations in its N‐terminal region, and Q8A0N4 (endo‐beta‐N‐acetylglucosaminidase F1) showed rotational flexibility in its N‐terminal E set domain. An additional potential application of this method is selecting the optimal conformation generated by different models. Supporting Information S1: Figures S10–S12 illustrated the performance of AFzero‐derived cross‐entropy versus AF2 confidence scores in selecting the best alternative protein conformations from AFsample2 candidates by plotting TM‐score deviations across top‐k selections. Systems where the best AFzero‐based scoring outperformed AF2 accounted for 22 of all 38 cases provided by AFsample2, except for MFSD2A_OUT. These findings support the broader applicability of our approach for probing hidden conformational landscapes in proteins. We anticipate that future improvements in computational efficiency and better decoy ranking approaches will further enable such investigations at scale.

## 4 CONCLUSIONS

In summary, this study explored the hidden multiple‐state structural information within AF2’s predictions without shallowing MSA and established an approach to incorporate alternative conformation signals derived from deep‐learning predictions into physics‐based modeling. We tested a wide range of structural variations and protein sizes, which demonstrated the effectiveness of our protocol. This framework can serve as a rapid hypothesis‐testing tool for exploring the likely alternative conformation of a protein structure from a given sequence. Our approach is orthogonal to current AF2‐based MSA‐subsampling methods and serves as a complementary strategy, leveraging deep MSAs to maintain relatively high structural fidelity while introducing structural diversity through distogram. Experimental techniques such as protein conjugation, nuclear magnetic resonance, and fluorescence resonance energy transfer can also be combined with our protocol to unravel the dynamics and functions of multi‐state proteins.

## 5 MATERIALS AND METHODS

### 5.1 Overall protocol

1. As shown in Figure 2, we extracted distograms from AF2’s predictions. These distograms served as the foundation for identifying residue pair distances associated with alternative conformations.
2. Starting from either state, we calculated its corresponding distogram and compared it to the AF2 prediction. Signals indicating alternative conformations were recorded in the alt‐distogram (distogram for alternative conformation).
3. We used the alt‐distogram for further structure modeling and designed the distance‐based energy restraint function that accounted for both alternative and common conformational signals.
4. We generated a series of structural models using Rosetta, incorporating the restraint energy function. These models underwent evaluation to give the best model.

### 5.2 Protein systems studied in this work

Our study utilized a dataset of 20 two‐state proteins, selected from databases curated by Flores et al. [48, 49, 50] and Saldaño et al. [13]. This diverse set of proteins exhibits various types of conformational changes (details in Supporting Information S1: Table S1), which we categorized as follows:

·
Hinge‐bending: These proteins display straightforward “open” and “closed” domain movements. Examples include LBP, glutamine‐binding protein (GlnBP), RBP, and ALBP.
·
Inter‐domain rotations: This category includes proteins such as DAHPS and 48G7g, which exhibit rotational movements between domains.
·
Single‐domain local rearrangements: Several proteins in our dataset show localized changes in helices and loops within a single domain. Examples are S100B, CNBD, NTRC, AP4A, S100A1, T4L, and NTnC. Also, there are some membrane proteins including sugar symporter (STP10), ZnT8, hydantoin transport protein (Mhp1), and ASCT2.
·
Complex multi‐domain rearrangements: Some proteins in our dataset, such as AK, GroEL, and HSPD1, exhibit a combination of different conformational changes across multiple domains.

Detailed information about all structures, downloaded from the PDB [47], is summarized in Supporting Information S1: Table S1.

### 5.3 Source of distogram

We used ColabFold v1.5.2 [3, 17] for structure predictions of all systems. To optimize accuracy and explore potential multi‐state conformations, we implemented the following parameter choices: (1) We chose MSA depth as *max_msa* = 512:1024 in the advanced settings; (2) We disabled recycling (*num_recycles* = 0) to focus on the initial predictions; (3) No templates were used in our predictions; (4) We used a single seed (*num_seeds* = 1), resulting in five models per prediction process; (5) We selected the “save_all” option, with particular focus on the “.pickle” file for distogram extraction. All other parameters adhered to ColabFold’s default settings.

AlphaFold’s algorithm converts pair representations into 64 distance bins [3], assigning probabilities using a softmax function. These bins span distances from 2 Å to 22 Å in equal increments, with the final bin encompassing all distances beyond 22 Å. To calculate the probability of distances between residue pairs, we processed the distogram logits data from the “.pickle” file using the softmax function Equation (1). This calculation was performed for $C_{\beta }$ atoms of all residue pairs, except for glycine, where we used the $C_{\alpha }$ atom due to the absence of a $C_{\beta }$.

(1) $$\text{softmax}\left({\text{DM}\_\text{logits}}_{i}\right)=\frac{\exp\left({\text{DM}\_\text{logits}}_{i}\right)}{\sum_{j}\exp\left({\text{DM}\_\text{logits}}_{j}\right)}$$

### 5.4 Extracting information on alternative conformations from distogram analysis

To identify potential alternative conformations in protein structure predictions, we developed a systematic approach for analyzing the complex features exhibited in the distograms. Our method focuses on detecting and classifying multi‐peak signals in the predicted distance‐probability distributions. The classification criteria and corresponding signal types are illustrated in Figure 3. Our analysis procedure consists of three major steps:

1. Initial peak detection and classification: For each residue pair i‐j, we examined the first 63 distance bins, reserving the last bin for long‐distance signal analysis. We identified local maxima and selected the top two (max1, max2). These peaks are checked based on their relative probabilities and separation: the probability sum of two distinct peaks should exceed 0.1; the probability of two maxima should satisfy $p_{\max\;2}\;>\;0.1p_{\max\;1}$; closely spaced peaks ($p_{\max\;1}-p_{\max\;2}\;<\;0.05p_{\max\;1}$) requiring consideration of a third maximum.
2. Bivariate Gaussian distribution fitting: For potential multi‐peak signals, we apply a bivariate Gaussian distribution fit (Equation (2)) with the following initial parameters. $\mu_{1},\mu_{2}$ are corresponding distances of two maxima, the initial amplitude $a_{1}={\;a}_{2\;}=0.1$, and standard deviations $\sigma_{1}=\sigma_{2\;}=1$.
(2) $$p\left(x\right)=\frac{a_{1}\;\exp\left(-\frac{{\left(x\;-\mu_{1}\right)}^{2}}{2\sigma_{1}^{2}}\right)}{\sigma_{1}\sqrt{2\pi }}+\frac{a_{2}\;\exp\left(-\frac{{\left(x\;-\mu_{2}\right)}^{2}}{2\sigma_{2}^{2}}\right)}{\sigma_{2}\sqrt{2\pi }}$$
  According to the fitting results, we classify the signals into the following categories:
  · Class I: Peaks with σ < 3, ensuring distinct distributions.
  · Class II: Secondary peak is weak but significantly above the inter‐peak saddle point ($\left(p_{\max\;2}-p_{\text{saddle}}\right)/p_{\max\;2\;}>\;0.05$).
  · Class III: Peak is well‐defined and falls within appropriate distance ranges (μ+σ<21.6875 Å).
3. Long‐distance signal analysis: We assess the probability of the last distance bin ($p_{\text{last}}$) to identify significant long‐distance signals. A notable but not dominant long‐distance signal is indicated when $p_{\text{last}}$ is larger than any fitted amplitude and falls between 0.1 and 0.8.

For distributions with a single main peak, we apply a univariate Gaussian fit and similar classification criteria. Multi‐peak cases involving long‐distance signals are classified as Class IV to VI, depending on the fitting results:

·
Class IV: Single peak from one maximum in step 1.
·
Class V: Only one peak fitted by Equation (2) in step 2.
·
Class VI: Peaks fitted in step 2 with σ > 3 are combined as one single peak.

Following this signal classification criteria, we further analyze the signals to identify and characterize alternative conformations:

- Peak analysis: For each residue pair, we determine the number of signal peaks ($n_{\text{peak}}$) and their corresponding breadths. Residue pairs with $n_{\text{peak}}\;>\;1$ are considered potential indicators of alternative conformations.
- Comparison with known structures: We compare predicted distograms with known structures to validate the signals.
  · For multi‐peak signals ($n_{\text{peak}}\;>\;1$), we remove the peak corresponding to the distance in the known structure and retain the remaining signals in alt‐distogram.
  · For single‐peak signals ($n_{\text{peak}}=1$), we compare the peak breadth with the known distance. If a discrepancy is found, we include this signal in our further modeling.
- Signal classification and precision calculation: We classify signals effective in predicting the alternative state into two categories, true positive multi‐peak signals (${\text{TP}}_{m}$) and true positive single‐peak signals (${\text{TP}}_{s}$). Both categories contribute to our final precision calculation Equation (3), where $C_{i}$ represents conformational state i.
(3) $$\text{Precision}\left(C_{i}\right)=\frac{{\text{TP}}_{m}+{\text{TP}}_{s\_C_{i}}}{{\text{TP}}_{m}+{\;\text{FP}}_{m}+{\;\text{TP}}_{s\_C_{i}}+{\text{FP}}_{s\_C_{i}}}$$
  When calculating the ratio of alternative signals for analysis, we normalize the number of alternative signals by the length of protein sequence.

### 5.5 Model building and assessment

#### 5.5.1 Structural modeling with Rosetta

Starting with one known protein structure, for example C1, we used Rosetta to build plausible models of the alternative conformation with restraints derived from the above distogram signals. Three functional modules of PyRosetta [51], pose initialization, minimization, and full‐atom refinement were employed in this process.

We initialized the pose using the “centroid” switch type. To accommodate our distance‐energy function, we mutated glycine residues to alanine, ensuring all residues had $C_{\beta }$ atoms for distance calculations.

To convert the predicted distance distributions into Rosetta energy restraints, we adapted the probability‐to‐energy transition method from trRosettaX [6]. Briefly, our restraint conversion protocol distinguishes between two types of signals, the alternative‐conformation signals and the common signals shared between two states. For the former, we gently flattened the energy barrier around the starting distance and then introduced a gradual slope that pulled the structure toward the alternative distance distribution. For the latter, we only slightly smoothed the highest energy peaks to allow some flexibility without enforcing a large change. The overall strength of the restraints for the common signals was adjusted by a scaling factor that took into account the ratio of alternative signals, the proportion of those signals at long range, and the normalized entropy of the distance distribution. These energy profiles were then added as distance restraints into the Rosetta scoring function, with a single weight parameter that controlled their influence during the structural search.

Below, we present our detailed approach to differentiating between residue pairs with alternative conformation signals and those with common distributions between two states:

1. For alternative conformation signals: As shown in Figure 4A,B, we flattened energy barriers at locations corresponding to the initial distance peak using a hyperbolic tangent function ($E_{ij\_\text{temp}}$, Equation (4)). We then applied gradient descent toward the new distribution ($E_{ij\_\text{alt}}$, Equation (5)) based on the initial distance between residue pairs ($d_{ij}$). Within the Equation (5), n is the bin index of the distance histogram (bins 1–64, covering 2–22 Å). d(n) is the distance value at bin n. Index $n_{0}$ identifies the bin that corresponds to the initial $C_{\beta }$ – $C_{\beta }$ distance $d_{ij}$ of the residue pair in the starting structure. Index $n_{1}$ is the first bin at which the original energy profile $E_{ij}\left(d\right)$ crosses zero. The linear term $\frac{n-n_{0}}{n_{1\;}-n_{0}}$ gradually reduces the energy barrier and biases the pair toward the alternative distance, whereas the hyperbolic tangent preprocessing prevents sharp energy spikes that could trap the structure.

(4) $$E_{ij\_\text{temp}}=\left\{\begin{array}{cc} \;\tanh\left(E_{ij}\right), & E_{ij}\;>\;0\\ \;E_{ij}, & E_{ij}\;<\;0 \end{array}\right.$$

(5) $$E_{ij\_\text{alt}\;}=\left\{\begin{array}{cc} \;\left(E_{ij\_\text{temp}}\left(n\right)\right)-\frac{n-n_{0}}{n_{1}-n_{0}}, & n∼\left(n_{0},n_{1}\right)\\ \;E_{ij\_\text{temp}}, & \text{else} \end{array}\right.$$

where

(6) $$d\left(n_{0}\right)=d_{ij}$$

(7) $$E_{ij}\left(d\left(n_{1}\right)\right)=0$$

2. For common distributions between two states: As shown in Figure 4C,D, we slightly flattened energy barriers above a platform value Equation (9), where Mo Equation (8) is the statistical mode of positive energy values.

(8) $$Mo=\text{mode}\left(E_{ij}\right)$$

(9) $$E_{ij\_\text{com}}=\left\{\begin{array}{cc} \;\tanh\left(E_{ij}-Mo\right), & E_{ij}\;>\;Mo\\ \;E_{ij}, & E_{ij}\;<\;Mo \end{array}\right.$$

To enhance the influence of alternative signals, we introduced a scaling parameter mag Equation (10) that considers $p_{nl}$ the ratio of alternative signals to protein length, Equation (11), $p_{\text{last}‐\text{bin}}$ the proportion of long‐distance signals, Equation (12), and $H_{\text{norm}}$ (normalized entropy, Equation (14)). Among these equations, $N_{\text{alt}}$ is the number of signals connected to alternative conformations; $N_{\text{last}‐\text{bin}}$ is the number of signals in the last bin, which represents a large distance separation exceeding 22 Å; H(p) is the entropy related to original probabilities p in all bins.

(10) $$\text{mag}=\left\{\begin{array}{cc} \;\frac{\max\left\{\min\left(E_{ij\_\text{alt}}\right)\right\}}{\min\left\{\min\left(E_{ij\_\text{com}}\right)\right\}}\times \left(\frac{1}{1+e^{{-p}_{\text{last}‐\text{bin}}}}\right)\times p_{nl}\times H_{\text{norm}}, & \text{if}\;p_{\text{last}‐\text{bin}}\geq 0.5\\ \;\frac{\text{mean}\left\{\min\left(E_{ij\_\text{alt}}\right)\right\}}{\text{mean}\left\{\min\left(E_{ij\_\text{com}}\right)\right\}}\times \left(\frac{1}{1+e^{-p_{\text{last}‐\text{bin}}}}\right)\times p_{nl}\times H_{\text{norm}}, & \text{if}{\;p}_{\text{last}‐\text{bin}}\;<\;0.5 \end{array}\right.$$

where

(11) $$p_{nl}=N_{\text{alt}}/L$$

(12) $$p_{\text{last}‐\text{bin}}=N_{\text{last}‐\text{bin}}/N_{\text{alt}}$$

(13) $$H\left(p\right)=-\sum_{i=1}^{64}p_{i}\cdot \ln\left(p_{i}\right)$$

(14) $$H_{\text{norm}}=\frac{H_{\max}-H}{H_{\max}-H_{\min}}$$

A higher value of $p_{nl}$ indicates a more extensive conformational change, which generally requires a different balance between the two sets of restraints. A high fraction of $p_{\text{last}‐\text{bin}}$ signals a substantial opening or widening motion, which is sterically more demanding and therefore requires careful tuning of the common‐signal restraint strength. When the prediction is sharp (low entropy), $H_{\text{norm}}$ is close to 1, strengthening the common restraints to help preserve the overall fold; when the prediction is diffuse (high entropy), $H_{\text{norm}}$ is low, which weakens the common restraints and gives the alternative signals more freedom to drive the structural change. The piecewise form of mag also uses the ratio of the minimum energies of the alternative and common signals, and applies a further sigmoidal modulation via $p_{\text{last}‐\text{bin}}$ to avoid over‐aggressive pulling when long‐range contacts dominate. Together, these factors enable the method to automatically adapt the restraint strengths according to the specific features of the predicted distograms. The final energy function for common distributions is: $E_{ij\_\text{com}\_\text{final}}=\text{mag}\times E_{ij\_\text{com}}$. This approach allows us to systematically model alternative protein conformations while accounting for the complex interplay between different structural signals. The opening or widening tendency between domains or helices is normally harder to achieve. By differentiating between alternative and common signals and applying appropriate scaling, we aim to achieve more accurate predictions of the alternative conformation.

During our Rosetta‐based modeling, 15 different parameter settings were employed, with each setting undergoing 10 independent sampling runs, resulting in a total of 150 independent samplings. The parameter design included the number of iterations for the RepeatMover loop, ncycle, with values ranging from 1 to 10, and the weight of the alt‐distogram‐based energy function, U, with values ranging from [0.02, 0.04, 0.06, 0.08, 0.1, 0.2, 0.4, 0.6, 0.8, 1.0, 1.2, 1.4, 1.6, 1.8, 2.0].

#### 5.5.2 Assessment of modeled structures

We evaluated the modeled structures using Rosetta’s energy score function, which comprises two components: $E_{\text{Rosetta}}$ and $E_{\text{Restraint}}$. $E_{\text{Rosetta}}$ is the sum of all energy terms in the ref2015 scoring function [52], commonly used to assess the plausibility of Rosetta‐built structures. Negative $E_{\text{Rosetta}}$ values indicate plausible structures. $E_{\text{Restraint}}$ represents the energy resulting from distance restraints between all residue pairs, specifically using the AtomPair restraint for distance‐based potentials ($E_{ij\_\text{alt}},E_{ij\_\text{com}\_\text{final}}$). We only considered models with negative $E_{\text{Restraint}}$ for further analysis, as positive values indicate failure to meet predicted distance restraints.

For each protein, we normalized the energy scores across 150 models sampled in one prediction using Equation (15). i represents each modeled structure, and $E_{\text{Rosetta}\_\max}$ and $E_{\text{Rosetta}\_\min}$ are the maximum and minimum energy scores, respectively.

(15) $$E_{\text{norm}}\left(i\right)=\frac{E_{\text{Rosetta}}\left(i\right)\;{-E}_{\text{Rosetta}\_\min}}{E_{\text{Rosetta}\_\max}-E_{\text{Rosetta}\_\min}}$$

Model selection criteria:

From the perspective of prediction, we developed a set of criteria to select the best‐scored structures based on energy scores and TM‐score differences (ΔTM) relative to the initial structure.

1. Eliminate the 10% of models with the worst $E_{\text{Restraint}}$ scores.
2. Retain models with $E_{\text{Rosetta}}\;<\;0$.
3. Rank models by $E_{\text{norm}}$, focusing on those with $E_{\text{norm}}\;<\;0.8\times \max\left(E_{\text{norm}}\right)$.
4. Calculate ΔTM ($\Delta TM=1-{\text{TM}}_{\text{initial}‐\text{relative}}$) and select the top 10 models with the largest ΔTM.
5. Choose the model with the lowest $E_{\text{norm}}$ among these 10 as the final best‐scored structure.

We used RMSD and TM‐score to assess the structural similarity between predicted models and the alternative structures solved experimentally. RMSD was calculated using the equation $\text{RMSD}=\sqrt{\frac{1}{N}\sum_{i=1}^{N\;}{\Delta r_{i}}^{2}}$ after aligning the $C_{\alpha }$ atoms of the two structures, where $\Delta r_{i}$ is the distance between each pair of corresponding atoms. TM‐score was calculated using TM‐align developed by Zhang Lab [53] to describe the topological similarity of conformations. Visualization of structures was performed using PyMOL.

## AUTHOR CONTRIBUTIONS

**Jiaxuan Li**: Methodology; software; formal analysis; investigation; data curation; visualization; writing—original draft. **Zefeng Zhu**: Formal analysis; investigation; validation; writing—original draft. **Chen Song**: Conceptualization; supervision; project administration; funding acquisition; writing—review and editing.

## ETHICS STATEMENT

This article does not contain any studies with human or animal subjects performed by any of the authors.

## CONFLICT OF INTEREST STATEMENT

The authors declare no conflicts of interest.

## Supporting information

Supporting Information S1

## Data Availability

The code and data can be accessed via the GitHub website (ComputBiophys/AltConfDist).
