# Supplementary material for: Predicting the alternative conformation of a known protein structure based on the distogram of AlphaFold2
Source: Quant Biol. 2026 Nov 6;14(4):e70062. doi: 10.1002/qub2.70062 (PMC13742378; doi:10.1002/qub2.70062)
Supplement: Supplementary file 1 [file QUB2-14-e70062-s001.pdf]

Supplementary Information for:

Predicting the alternative conformation of a known protein structure based on the distogram of AlphaFold2

Jiaxuan Li[1,2], Zefeng Zhu[1,3], and Chen Song[*,1,3]

[1]Center for Quantitative Biology, Academy for Advanced Interdisciplinary Studies, Peking University, Beijing 100871, China

[2]College of Life Sciences, Yantai University, Yantai 264005, China

[3]Peking-Tsinghua Center for Life Sciences, Academy for Advanced Interdisciplinary Studies, Peking University, Beijing 100871, China

[*]E-mail: c.song@pku.edu.cn

Supplementary Tables

Table S1: Details of the 20 protein systems analyzed in this study. PDB IDs for the experimental structures of conformation 1 (C1) and conformation 2 (C2) are provided. The penultimate column lists the sequence lengths of the aligned structures for the two states. The final column summarizes the types of conformational changes. The suffix of the PDB ID indicates the serial number of the selected NMR model, while _A/_B denotes the chain identifier.

Protein	Name	C1_ID	C2_ID	Aligned sequence length	Type of conformational change
**T4L**	T4 Lysozyme	172L_A	2LZM_A	161	single domain helices & loops
**AK**	Adenylate kinase	4AKE_B	2ECK_B	214	combination of changes in multi-domains
**LBP**	Leucine binding protein	1USG_A	1USI_A	345	hinge-bending
**ALBP**	d-allose binding protein	1GUD_A	1RPJ_A	288	hinge-bending
**RBP**	Ribose binding protein	1URP_A	2DRI_A	271	hinge-bending
**GroEL**	60 kDa chaperonin	1AON_A	4HEL_A	524	combination of changes in multi-domains
**GlnBP**	Glutamine binding protein	1GGG_A	1WDN_A	220	hinge-bending
**STP10**	Sugar transport protein 10	7AAQ_A	7AAR_A	480	transmembrane helices & loops
**DAHPS**	DAHP synthase	1VR6_A	1RZM_A	338	domain rotation
**S100B**	S100 calcium-binding protein	1SYM-2_B	1XYD-15_A	83	single domain helices & loops
**CNBD**	Nucleotide-activated K^+ channel binding protein	2KXL-8_A	2K0G-9_A	142	single domain helices & loops
**HSPD1**	Human mitochondrial chaperonin	7AZP_A	4PJ1_A	524	combination of changes in multi-domains
**NTnC**	Regulatory domain of Troponin C	1SKT-10_A	1TNQ-33_A	90	single domain helices & loops
**ZnT8**	Zinc transporter 8	6XPF_A	6XPD_A	288	transmembrane helices & loops
**NTRC**	Receiver domain of transcriptional enhancer binding protein	1NTR-7_A	1KRX-4_A	124	single domain helices & loops
**Mhp1**	Hydantoin transport protein	2JLN_A	2X79_A	463	transmembrane helices & loops
**ASCT2**	Neutral amino acid transporter	6GCT_A	7BCQ_A	427	transmembrane helices & loops
**AP4A**	Ap4A hydrolase	1F3Y-17_A	1JKN-11_A	165	single domain helices & loops
**S100A1**	S100 metal binding protein	1K2H-2_A	1ZFS-13_B	93	single domain helices & loops
**48G7g**	Immunoglobulin 48G7 germline Fab	2RCS_H	1AJ7_H	217	domain rotation

Table S2: Evaluation of control experiment 1 (CTRL1) using the entire distogram predicted by the default AF2 for modeling the 20 selected proteins. The superscripts, C1 and C2, indicate the conformations relative to which the TM-scores were calculated. Successful predictions are highlighted in bold.

Protein	TM$_{2states}$	TM-scores (C1 to C2)				TM-scores (C2 to C1)			
		Best scored^{C1}	Best scored^{C2}	Best sampled^{C1}	Best sampled^{C2}	Best scored^{C1}	Best scored^{C2}	Best sampled^{C1}	Best sampled^{C2}
**AK**	**0.69**	0.74	**0.90**	0.73	**0.91**	0.74	0.90	0.74	0.90
**LBP**	**0.64**	0.86	0.75	0.83	0.77	0.66	0.99	0.67	0.99
**ALBP**	**0.76**	0.78	**0.95**	0.79	**0.95**	0.78	0.95	0.79	0.95
**RBP**	**0.76**	0.78	**0.99**	0.78	**0.99**	0.78	0.99	0.79	0.99
**GroEL**	**0.64**	0.64	**0.78**	0.64	**0.82**	0.66	0.75	0.69	0.77
**GlnBP**	**0.67**	0.84	0.70	0.86	0.71	0.66	0.98	0.67	0.98
**STP10**	**0.82**	0.84	**0.93**	0.88	**0.93**	0.84	0.95	0.85	0.95
**DAHPS**	**0.81**	0.84	0.81	0.84	0.81	**0.83**	0.81	**0.84**	0.81
**S100B**	**0.55**	0.60	**0.82**	0.60	**0.82**	0.60	0.84	0.61	0.84
**CNBD**	**0.74**	0.71	**0.86**	0.71	**0.87**	0.71	0.98	0.71	0.85
**NTRC**	**0.64**	0.68	**0.75**	0.68	**0.76**	0.68	0.75	0.69	0.75
**AP4A**	**0.80**	0.84	**0.86**	0.83	**0.87**	0.83	0.87	0.83	0.87
**48G7g**	**0.57**	0.57	**0.81**	0.57	**0.82**	0.60	0.79	0.62	0.80
**S100A1**	**0.50**	0.51	**0.72**	0.52	**0.73**	0.51	0.73	0.53	0.74
**T4L**	**0.76**	0.97	0.80	0.97	0.81	**0.97**	0.80	**0.97**	0.80
**NTnC**	**0.55**	0.63	**0.74**	0.62	**0.74**	0.63	0.72	0.64	0.73
**HSPD1**	**0.64**	0.66	**0.74**	0.67	**0.75**	0.62	0.78	0.64	0.80
**ZnT8**	**0.70**	0.83	0.82	0.83	0.82	**0.83**	0.82	**0.83**	0.81
**Mhp1**	**0.88**	0.96	0.89	0.96	0.89	**0.95**	0.88	**0.96**	0.89
**ASCT2**	**0.65**	0.63	0.53	0.97	0.65	0.56	0.55	0.67	0.82

Table S3: Evaluation of control experiment 2 (CTRL2) without using the scaling parameter 'mag' in the energy functions for modeling the 20 selected proteins. The superscripts, C1 and C2, indicate the conformations relative to which the TM-scores were calculated. Successful predictions are highlighted in bold.

Protein	TM$_{2states}$	TM-scores (C1 to C2)				TM-scores (C2 to C1)			
		Best scored^{C1}	Best scored^{C2}	Best sampled^{C1}	Best sampled^{C2}	Best scored^{C1}	Best scored^{C2}	Best sampled^{C1}	Best sampled^{C2}
**AK**	**0.69**	0.72	**0.91**	0.72	**0.92**	0.77	0.87	0.79	0.85
**LBP**	**0.64**	0.84	0.78	0.78	**0.84**	0.67	0.99	0.67	0.99
**ALBP**	**0.76**	0.78	**0.95**	0.79	**0.95**	0.79	0.95	0.79	0.95
**RBP**	**0.76**	0.78	**0.99**	0.78	**0.99**	0.78	0.99	0.78	0.99
**GroEL**	**0.64**	0.78	0.63	0.82	0.64	**0.78**	0.69	**0.78**	0.70
**GlnBP**	**0.67**	0.85	0.72	0.83	0.74	0.66	0.98	0.67	0.98
**STP10**	**0.82**	0.90	**0.93**	0.90	**0.93**	**0.92**	0.91	**0.92**	0.91
**DAHPS**	**0.81**	0.83	0.81	0.84	0.81	**0.83**	0.81	**0.85**	0.81
**S100B**	**0.55**	0.60	**0.83**	0.60	**0.83**	0.60	0.83	0.61	0.84
**CNBD**	**0.74**	0.71	**0.87**	0.71	**0.87**	0.71	0.84	0.71	0.85
**NTRC**	**0.64**	0.68	**0.75**	0.68	**0.76**	0.68	0.75	0.69	0.75
**AP4A**	**0.80**	0.83	**0.88**	0.83	**0.88**	0.83	0.87	0.83	0.87
**48G7g**	**0.57**	0.57	**0.81**	0.57	**0.84**	0.59	0.79	0.61	0.80
**S100A1**	**0.50**	0.51	**0.73**	0.51	**0.74**	0.51	0.75	0.52	0.74
**T4L**	**0.76**	0.96	0.80	0.97	0.81	**0.96**	0.80	**0.97**	0.80
**NTnC**	**0.55**	0.63	**0.72**	0.62	**0.74**	0.63	0.73	0.64	0.73
**HSPD1**	**0.64**	0.66	**0.71**	0.66	**0.75**	0.64	0.78	0.64	0.78
**ZnT8**	**0.70**	0.82	**0.83**	0.82	**0.83**	**0.83**	0.79	**0.83**	0.80
**Mhp1**	**0.88**	0.96	0.90	0.95	0.90	**0.95**	0.88	**0.96**	0.88
**ASCT2**	**0.65**	0.62	0.53	0.98	0.65	0.57	0.55	0.68	0.77

Table S4: Additional 20 protein systems with one single state deposited in PDB.

Reference PDB	Name	UniProt
7PSJ_A	Beta-glucuronidase	C1F2K5
7PXL_A	Auxiliary activity 9	A0A0S2GKZ1
7YXJ_A	GH14974p	Q9VU77
7UQY_A	Exostosin-1	Q16394
7SEW_A	Histidine N-alpha-methyltransferase	P9WN47
7NNF_A	Ornithine carbamoyltransferase	P9WIT9
7W5T_A	nonheme iron and alpha-ketoglutarate dependent halogenase	A0A1U8X168
7WJA_A	Alpha-xylosidase	A2RM80
7RC6_A	Methyltransferase family protein	A0A2S0PEL5
7OK9_A	Penicillin-binding protein 1	A0A0H2WVW5
7VPG_A	mRNA export factor	P78406
7S4K_A	Particulate methane monooxygenase alpha subunit	G1UBD1
7SK4_A	Atypical chemokine receptor 3	P25106
7XQ1_A	Reduced folate transporter	P41440
7Q6Z_A	Protein-cysteine N-palmitoyltransferase HHAT	Q5VTY9
7NWF_A	Endo-beta-N-acetylglucosaminidase F1	Q8A0N4
7PBR_A	Holliday junction ATP-dependent DNA helicase RuvB	Q5M2B1
7VFD_A	Scaffold protein D13	P68440
7VDQ_A	Cyclin-dependent-like kinase 5	Q00535
7WRE_A	Transient receptor potential cation channel subfamily M member 8	Q8R4D5

Supplementary Figures

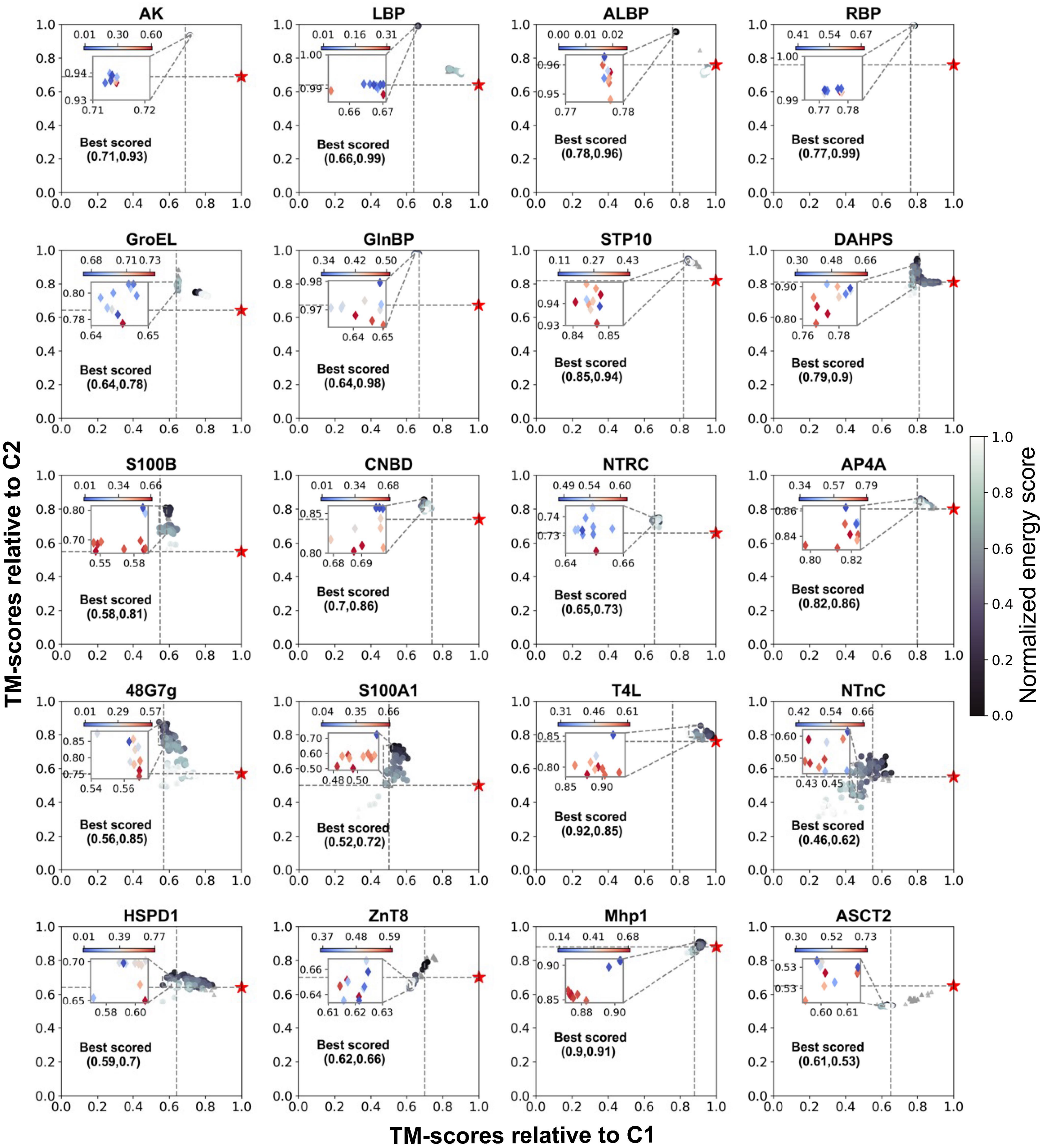

Figure S1: Rosetta sampling results for protein conformation prediction from C1 to C2. The initial structure (red star) and 150 models are shown, color-coded by normalized energy score (darker indicates lower energy). The dotted line represents the initial conformational similarity ($TM_{2states}$) between states. Grey triangles denote structures not satisfying the designed restraints. Effective samples tend to shift across the diagonal. Inset: Top 10 models based on selection criteria, with normalized energy scores ranging from blue (lowest) to red (highest). The blue point indicates the best-scored model; its TM-scores relative to C1 and C2 are displayed in each panel.

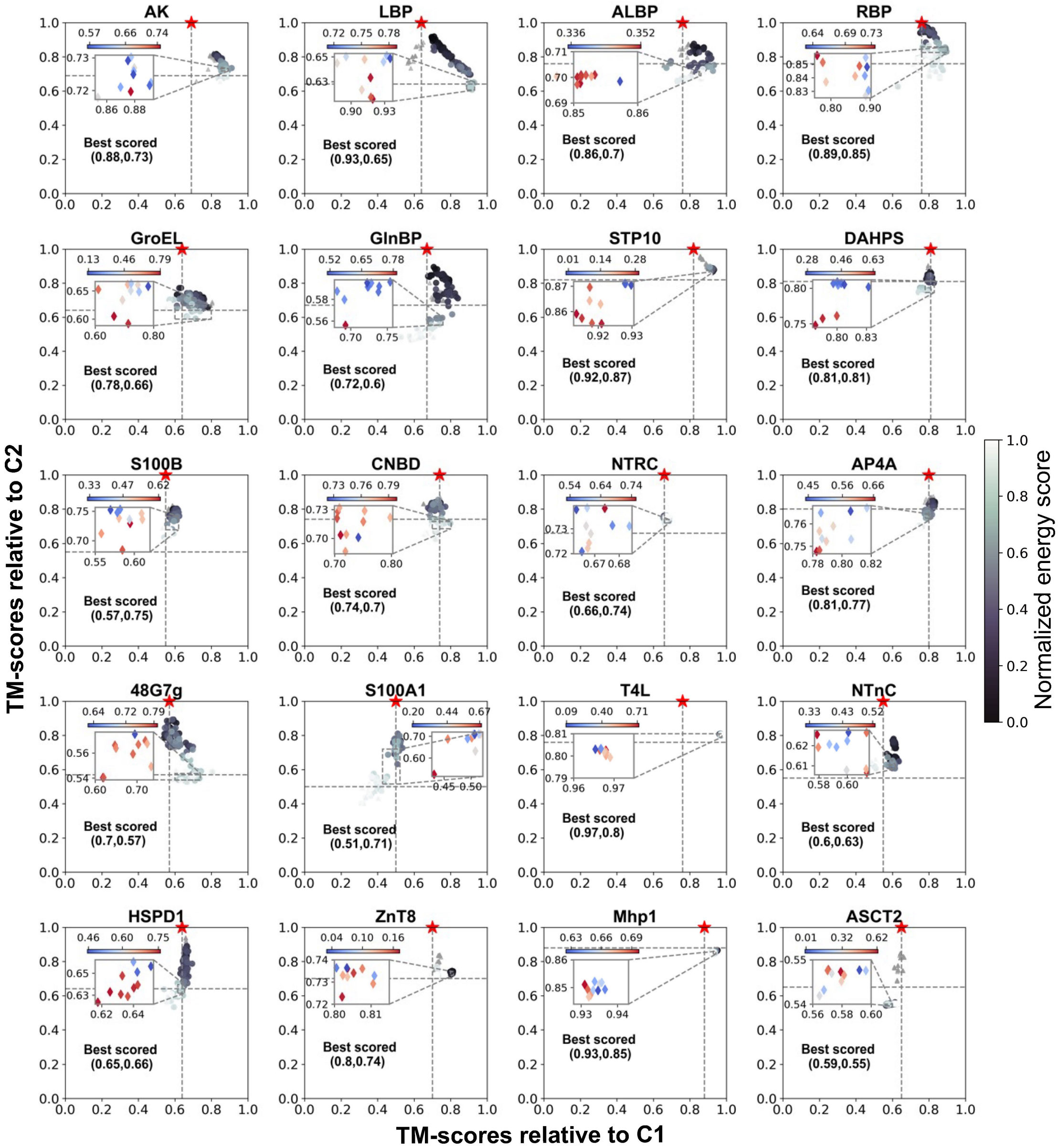

Figure S2: Rosetta sampling results for protein conformation prediction from C2 to C1. The initial structure (red star) and 150 models are shown, color-coded by normalized energy score (darker indicates lower energy). The dotted line represents the initial conformational similarity ($TM_{2states}$) between states. Grey triangles denote structures not satisfying the designed restraints. Effective samples tend to shift across the diagonal. Inset: Top 10 models based on selection criteria, with normalized energy scores ranging from blue (lowest) to red (highest). The blue point indicates the best-scored model; its TM-scores relative to C1 and C2 are displayed in each panel.

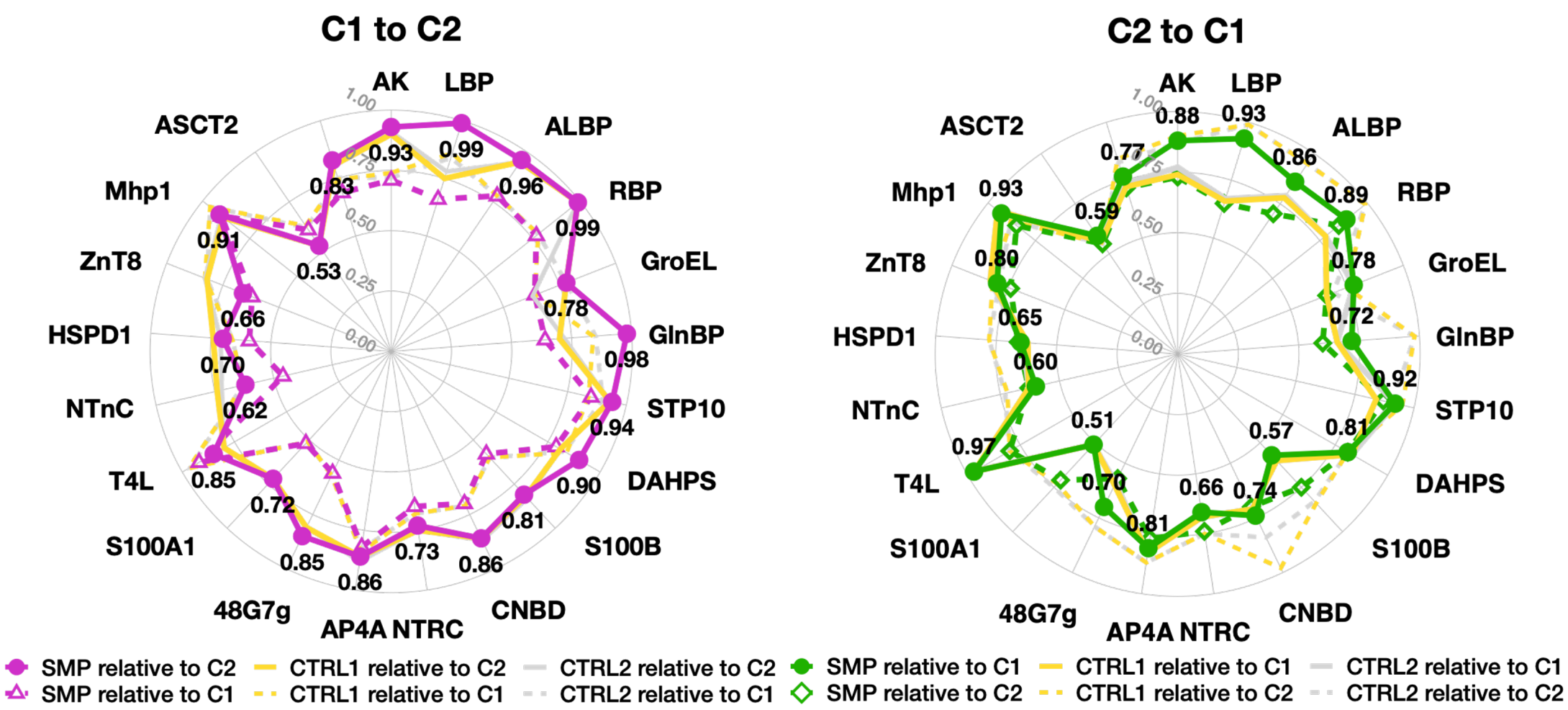

Figure S3: Radar maps comparing TM-scores of best-scored samples (SMP) with two control experiments. Control experiment 1 (CTRL1, yellow): sampling with the entire AF2-predicted distogram; control experiment 2 (CTRL2, grey): sampling without the scaling parameter 'mag' in energy functions. Solid lines represent TM-scores relative to the target state; dotted lines indicate TM-scores relative to the initial known state. Effective prediction rates for the second state are 85% (C1 to C2) and 70% (C2 to C1), where predicted models are more similar to the target state and further from the initial state. Control experiments show lower success rates: 65% and 20% for the two directions, respectively. The peripheral distribution of dotted lines indicates that structures often remain close to the initial state in the control sampling.

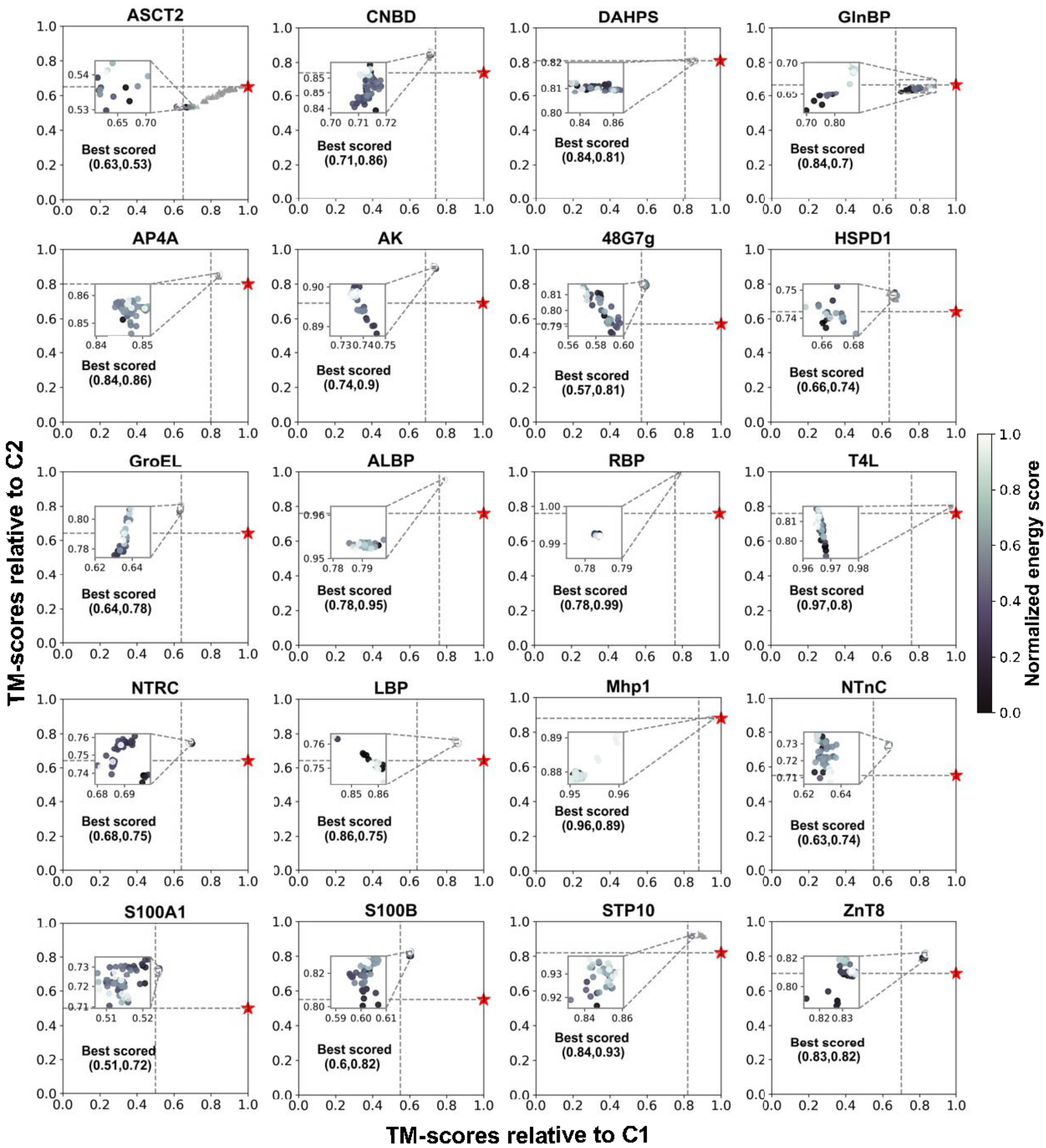

Figure S4: Rosetta sampling results for control experiment 1 (CTRL1). The figure displays the outcomes of Rosetta sampling using the full distogram predicted by default AlphaFold2 (AF2) from C1 to C2. The initial structure is marked with a red star, while 150 generated models are color-coded according to their normalized energy scores (darker colors represent lower energy). The dotted line illustrates the initial conformational similarity between states ($TM_{2states}$). Grey triangles denote structures that do not meet the designed restraints. The inset provides a zoomed-in view of clustered models, highlighting the best-scored model, which is the predicted structure with the lowest energy score. The TM-scores relative to C1 and C2 are shown in each panel.

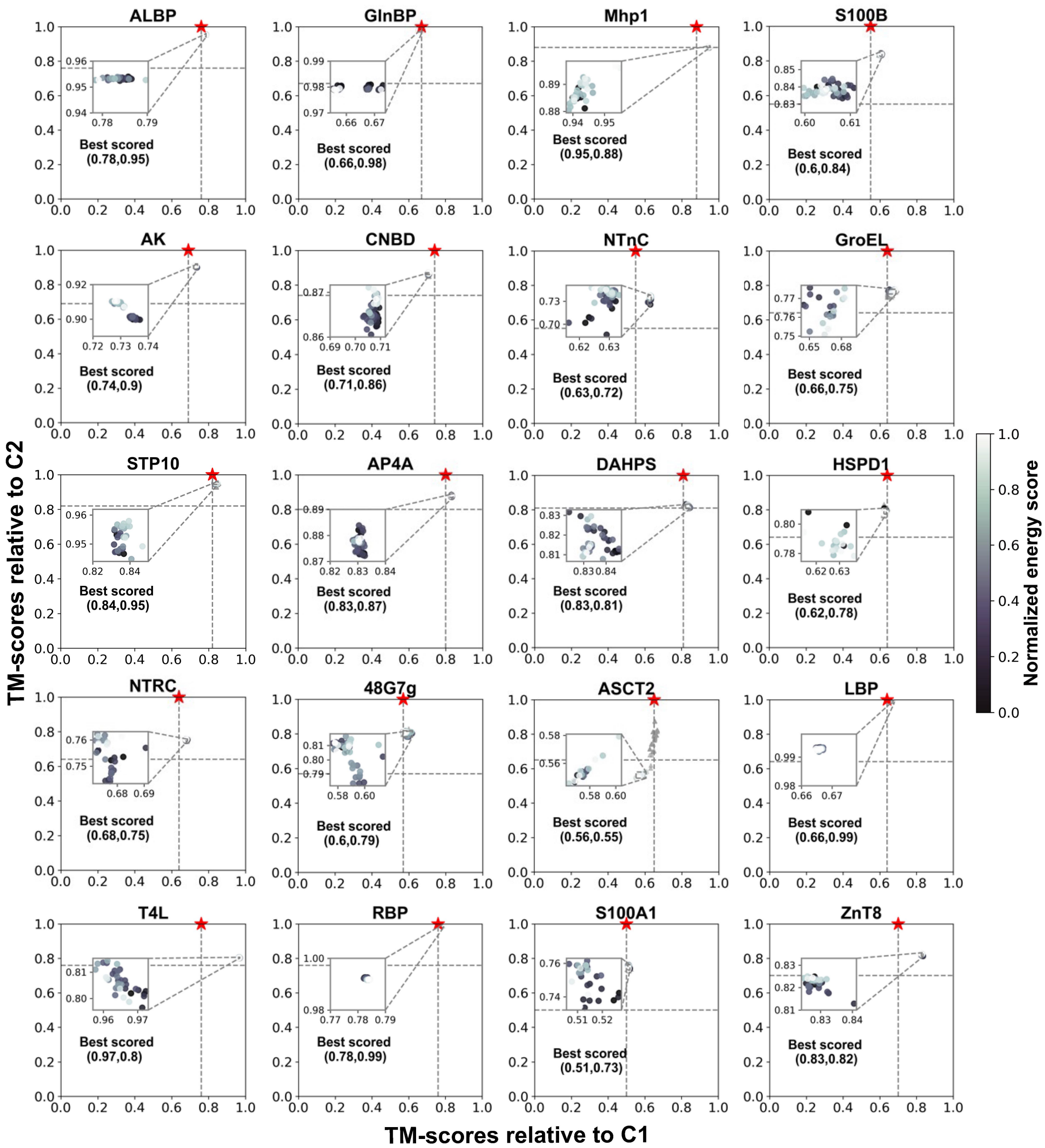

Figure S5: Rosetta sampling results for control experiment 1 (CTRL1). The figure displays the outcomes of Rosetta sampling using the full distogram predicted by default AlphaFold2 (AF2) from C2 to C1. The initial structure is marked with a red star, while 150 generated models are color-coded according to their normalized energy scores (darker colors represent lower energy). The dotted line illustrates the initial conformational similarity between states ($TM_{2states}$). Grey triangles denote structures that do not meet the designed restraints. The inset provides a zoomed-in view of clustered models, highlighting the best-scored model, which is the predicted structure with the lowest energy score. The TM-scores relative to C1 and C2 are shown in each panel.

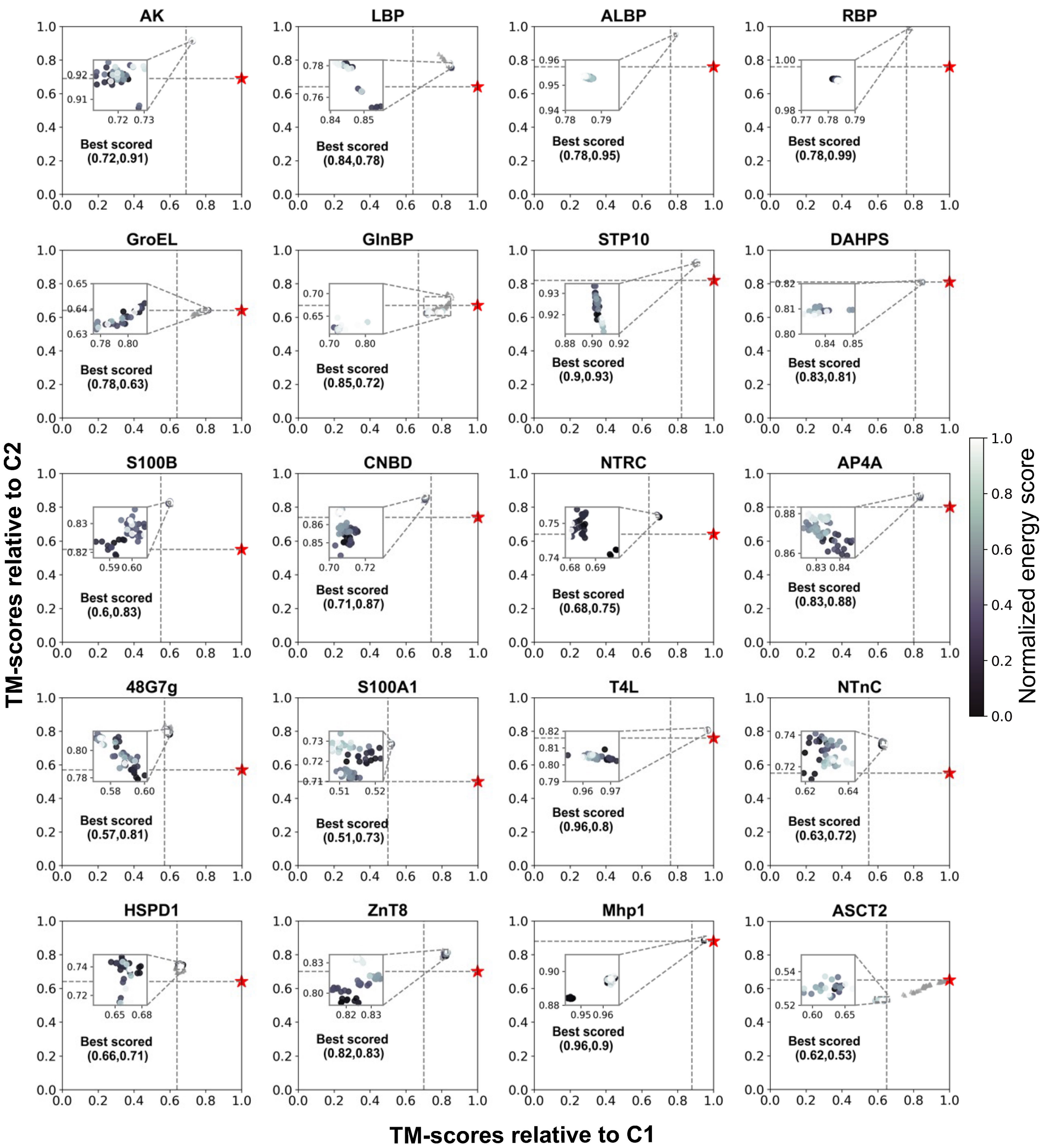

Figure S6: Rosetta sampling results for control experiment 2 (CTRL2). This figure presents the results of Rosetta sampling conducted without applying the scaling factor 'mag' in the energy functions during the transition from C1 to C2. The initial structure is denoted by a red star, and 150 generated models are displayed, with colors representing their normalized energy scores (darker colors indicate lower energy). The dotted line signifies the initial conformational similarity between states ($TM_{2states}$). Grey triangles denote structures that failed to satisfy the designed restraints. The inset offers a zoomed-in view of the clustered models, with a focus on the best-scored model – the predicted structure with the lowest energy score. The TM-scores relative to C1 and C2 are shown in each panel.

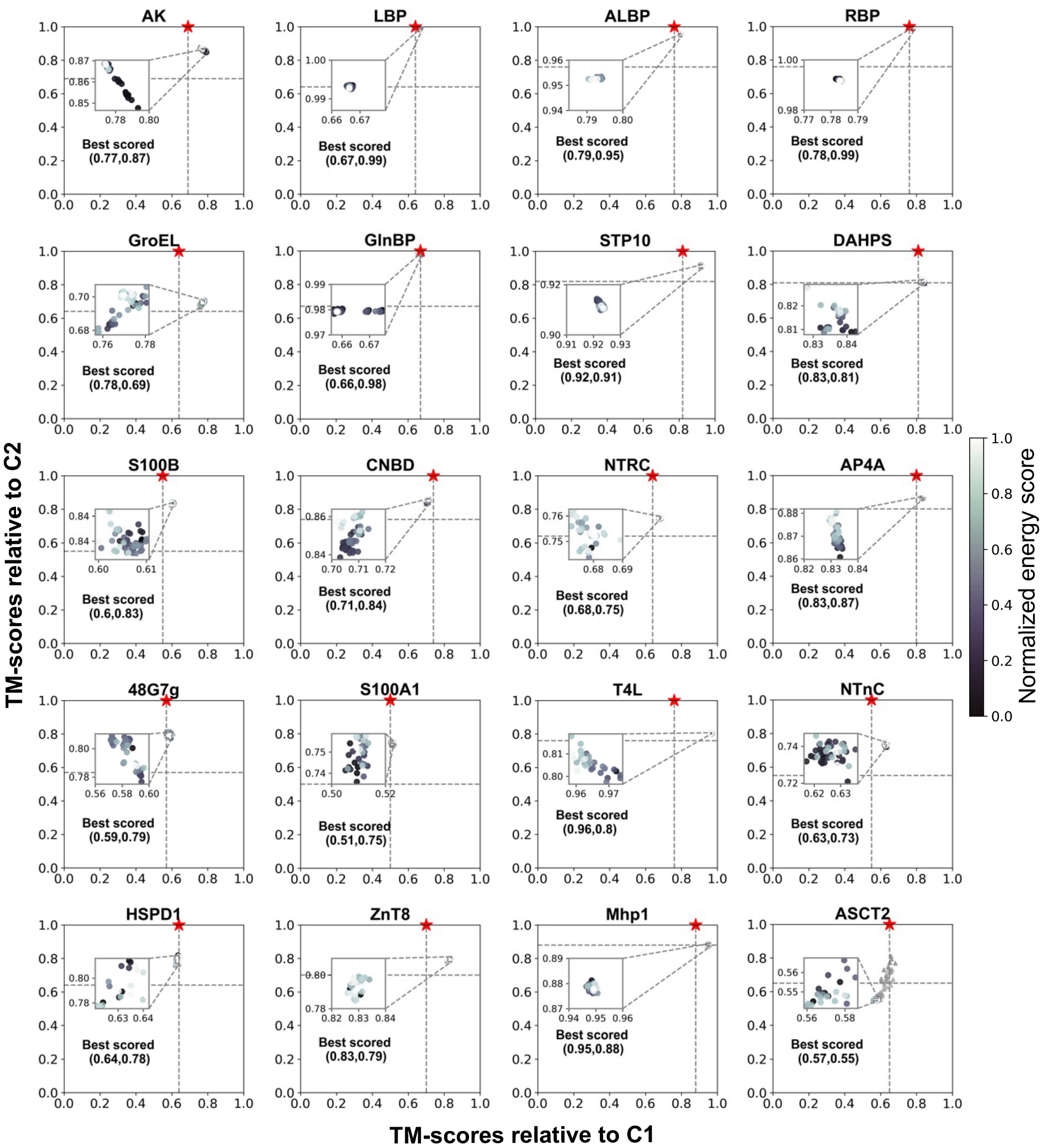

Figure S7: Rosetta sampling results for control experiment 2 (CTRL2). This figure presents the results of Rosetta sampling conducted without applying the scaling factor 'mag' in the energy functions during the transition from C2 to C1. The initial structure is denoted by a red star, and 150 generated models are displayed, with colors representing their normalized energy scores (darker colors indicate lower energy). The dotted line signifies the initial conformational similarity between states ($TM_{2states}$). Grey triangles denote structures that failed to satisfy the designed restraints. The inset offers a zoomed-in view of the clustered models, with a focus on the best-scored model – the predicted structure with the lowest energy score. The TM-scores relative to C1 and C2 are shown in each panel.

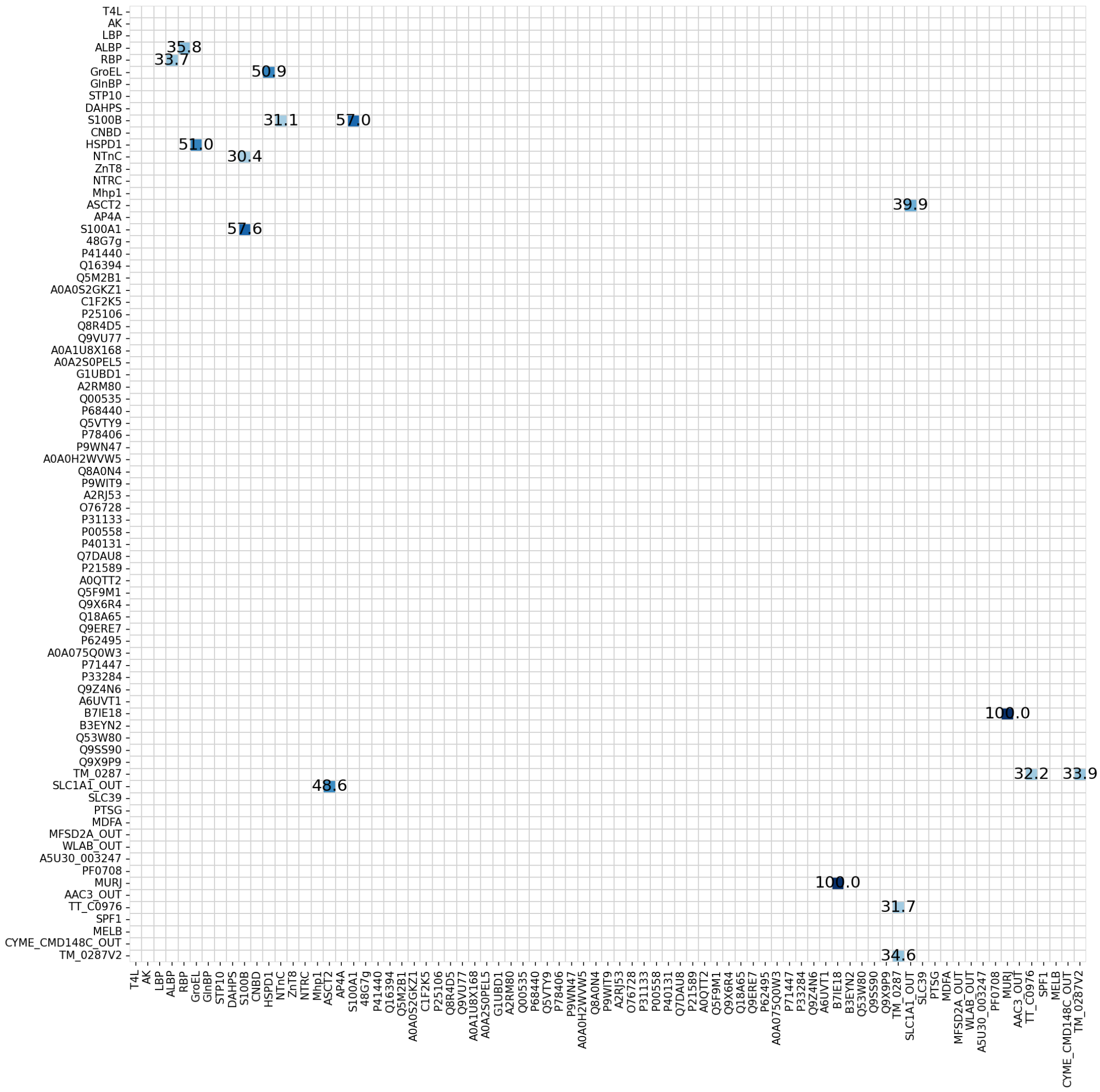

Figure S8: The pairwise sequence identity matrix of the protein systems studied in this work. Only the sequence identities above 30% are shown. The sequence identity values in each row are calculated with respect to the sequence in the column.

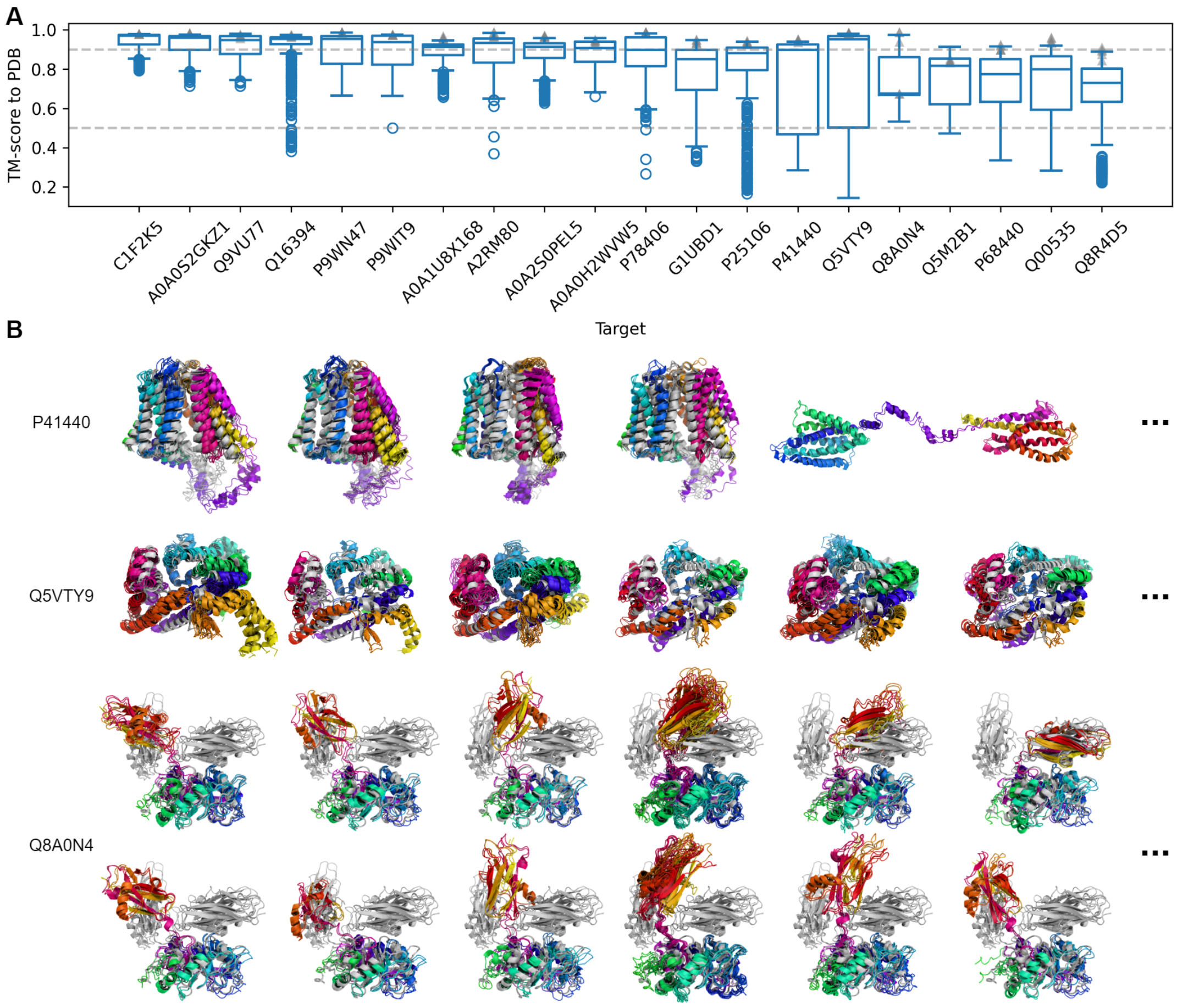

Figure S9: Five AFzero and 750 Rosetta samples for additional 20 protein systems with only one state deposited in PDB. (A) Boxplots show TM-score distributions of 750 Rosetta-generated structures compared to the known state in PDB for each protein system, sorted by average TM-score. TM-scores of five AFzero models are overlaid as gray triangles. (B) Representative examples of protein systems in which Rosetta-based sampling suggests potential alternative conformational states. AFzero models are depicted in gray, while Rosetta samples are shown in rainbow colors. Rosetta samples shown were clustered using Foldseek with a TM-score threshold of 0.9.

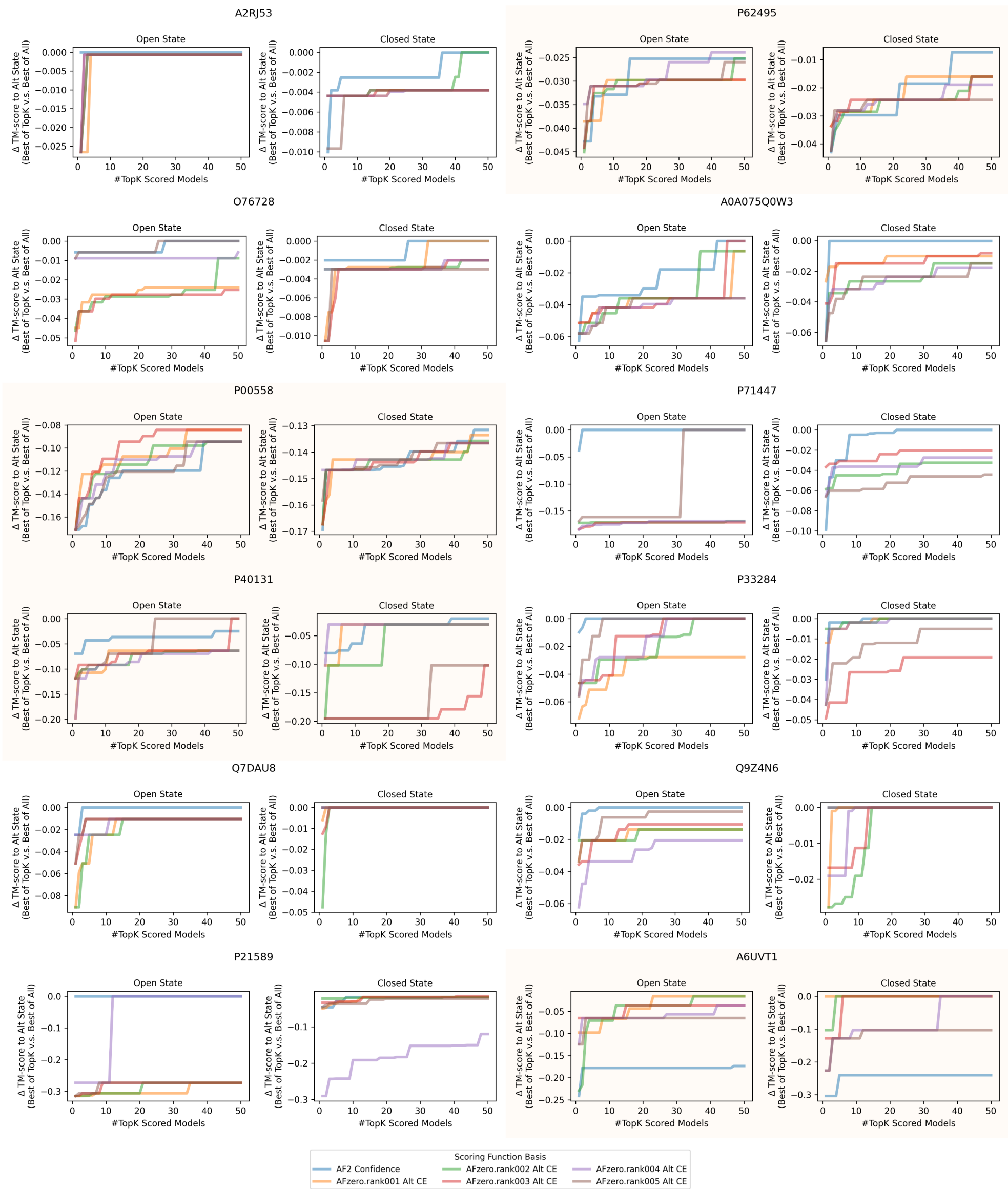

Figure S10: The performance of different scoring functions on selecting the best candidate of alternative conformations (part I). For each k, the TM-score deviation (the ground-truth alternative state as reference structure) between the best candidate within the top-k set of a particular scoring function and the best of all candidates, are shown. Besides the confidence score provided by AlphaFold2, the cross entropy between alternative distograms derived from AFzero and candidate models can also serve as scoring functions for selection. Protein systems for which the AFzero-based scoring function outperformed the AlphaFold2 confidence-based function in discriminating one of the alternative states are highlighted in light orange. The candidate models are from AFsample2's Zenodo dataset.

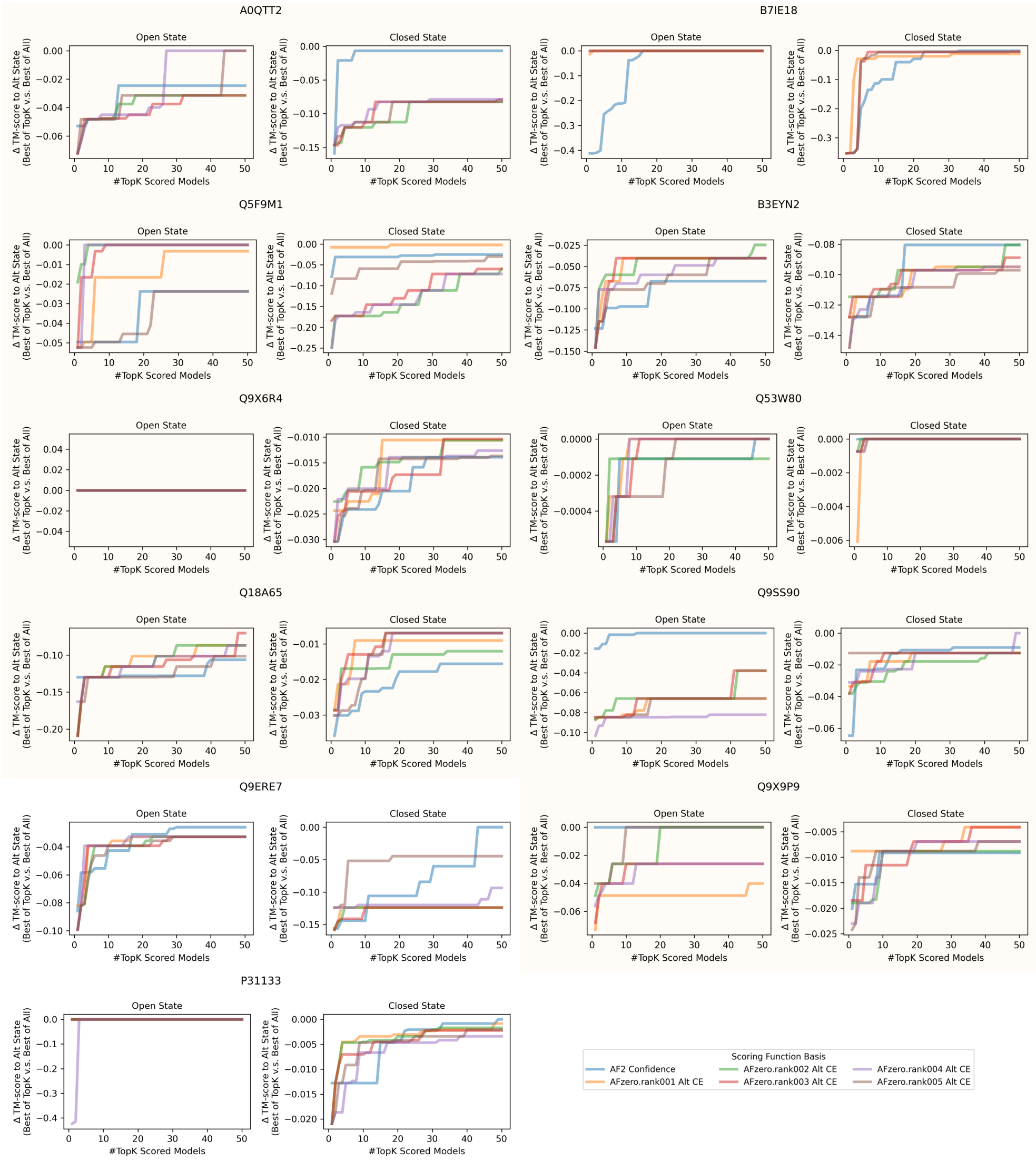

Figure S11: The performance of different scoring functions on selecting the best candidate of alternative conformations (part II). For each k, the TM-score deviation (the ground-truth alternative state as reference structure) between the best candidate within the top-k set of a particular scoring function and the best of all candidates, are shown. Besides the confidence score provided by AlphaFold2, the cross entropy between alternative distograms derived from AFzero and candidate models can also serve as scoring functions for selection. Protein systems for which the AFzero-based scoring function outperformed the AlphaFold2 confidence-based function in discriminating one of the alternative states are highlighted in light orange. The candidate models are from AFsample2's Zenodo dataset.

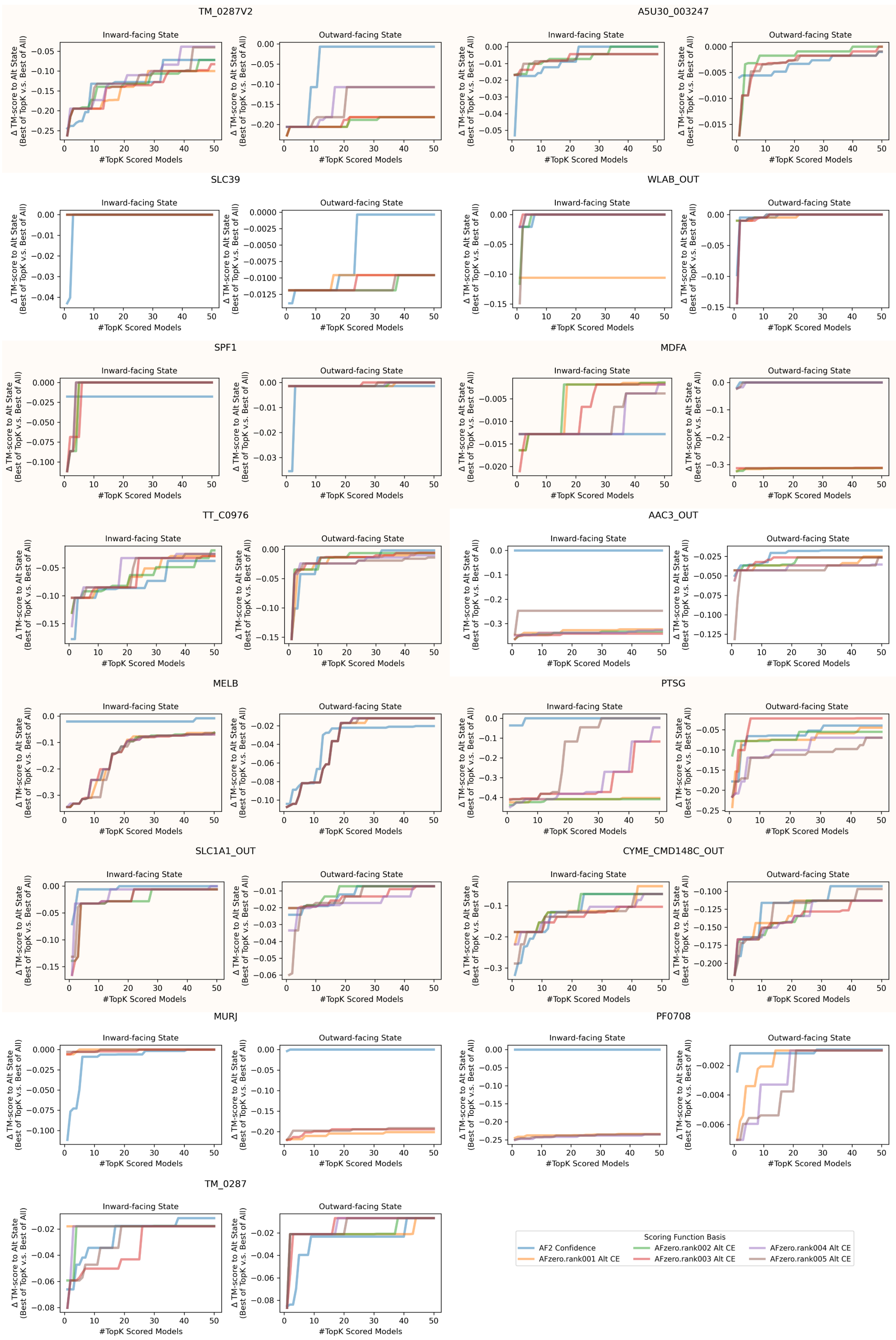

Figure S12: The performance of different scoring functions on selecting the best candidate of alternative conformations (part III). For each k, the TM-score deviation (the ground-truth alternative state as reference structure) between the best candidate within the top-k set of a particular scoring function and the best of all candidates, are shown. Besides the confidence score provided by AlphaFold2, the cross entropy between alternative distograms derived from AFzero and candidate models can also serve as scoring functions for selection. Protein systems for which the AFzero-based scoring function outperformed the AlphaFold2 confidence-based function in discriminating one of the alternative states are highlighted in light orange. The candidate models are from AFsample2's Zenodo dataset.
